# Genetic architecture of variation in heading date among Asian rice accessions

**DOI:** 10.1186/s12870-015-0501-x

**Published:** 2015-05-08

**Authors:** Kiyosumi Hori, Yasunori Nonoue, Nozomi Ono, Taeko Shibaya, Kaworu Ebana, Kazuki Matsubara, Eri Ogiso-Tanaka, Takanari Tanabata, Kazuhiko Sugimoto, Fumio Taguchi-Shiobara, Jun-ichi Yonemaru, Ritsuko Mizobuchi, Yusaku Uga, Atsunori Fukuda, Tadamasa Ueda, Shin-ichi Yamamoto, Utako Yamanouchi, Toshiyuki Takai, Takashi Ikka, Katsuhiko Kondo, Tomoki Hoshino, Eiji Yamamoto, Shunsuke Adachi, Hideki Nagasaki, Ayahiko Shomura, Takehiko Shimizu, Izumi Kono, Sachie Ito, Tatsumi Mizubayashi, Noriyuki Kitazawa, Kazufumi Nagata, Tsuyu Ando, Shuichi Fukuoka, Toshio Yamamoto, Masahiro Yano

**Affiliations:** National Institute of Agrobiological Sciences, 2-1-2 Kannondai, 305-8602 Tsukuba, Ibaraki Japan; Institute of the Society for Techno-innovation of Agriculture, Forestry and Fisheries, 446-1 Ippaizuka, Kamiyokoba, 305-0854 Tsukuba, Ibaraki Japan

**Keywords:** *Oryza sativa* L, Heading date, QTL, Natural variation, Genetic architecture

## Abstract

**Background:**

Heading date, a crucial factor determining regional and seasonal adaptation in rice (*Oryza sativa* L.), has been a major selection target in breeding programs. Although considerable progress has been made in our understanding of the molecular regulation of heading date in rice during last two decades, the previously isolated genes and identified quantitative trait loci (QTLs) cannot fully explain the natural variation for heading date in diverse rice accessions.

**Results:**

To genetically dissect naturally occurring variation in rice heading date, we collected QTLs in advanced-backcross populations derived from multiple crosses of the *japonica* rice accession Koshihikari (as a common parental line) with 11 diverse rice accessions (5 *indica*, 3 *aus*, and 3 *japonica*) that originate from various regions of Asia. QTL analyses of over 14,000 backcrossed individuals revealed 255 QTLs distributed widely across the rice genome. Among the detected QTLs, 128 QTLs corresponded to genomic positions of heading date genes identified by previous studies, such as *Hd1*, *Hd6*, *Hd3a*, *Ghd7*, *DTH8*, and *RFT1*. The other 127 QTLs were detected in different chromosomal regions than heading date genes.

**Conclusions:**

Our results indicate that advanced-backcross progeny allowed us to detect and confirm QTLs with relatively small additive effects, and the natural variation in rice heading date could result from combinations of large- and small-effect QTLs. We also found differences in the genetic architecture of heading date (flowering time) among maize, Arabidopsis, and rice.

**Electronic supplementary material:**

The online version of this article (doi:10.1186/s12870-015-0501-x) contains supplementary material, which is available to authorized users.

## Background

Many plant species are able to flower in the seasons best suited to their reproduction. This ability depends mainly on the accurate measurement of seasonal changes in day length and temperature [1,2]. Rice is a short-day plant, i.e. it requires a photoperiod shorter than a critical day length for heading and flowering to occur [3].

During last two decades, considerable progress has been made in our understanding of the molecular regulation of heading date in rice [4-9]. Rice photoperiodic flowering is controlled by two independent signaling pathways. The *OsGI*–*Hd1*–*Hd3a* pathway (*rice GIGANTEA*, *Heading date 1*, and *Heading date 3a*) is evolutionarily conserved in rice, as is the *GI*–*CO*–*FT* pathway (*GIGANTEA*, *CONSTANS*, and *FLOWERING LOCUS T*) in Arabidopsis. *Hd1* was the first heading date QTL cloned on the basis of natural variation in rice accessions [10]. *Hd1*, a homolog of Arabidopsis *CO*, promotes heading under short-day length (SD) conditions and represses it under long-day length (LD) conditions. *Hd1* promotes *Hd3a* expression under SD conditions, but inhibits *Hd3a* expression under LD conditions [11]. The repression of heading by Hd1 under LD conditions is enhanced by the kinase activity of Hd6 (Heading date 6), which is the α-subunit of casein kinase 2 [12,13]. Hd3a functions as a florigen [14]. Another florigen gene, *RFT1* (*Rice flowering locus T 1*), is a tandemly duplicated paralog of *Hd3a* [15]. *RFT1* expression increases under LD conditions, indicating that RFT1 is an LD-specific florigen [16,17]. The other signaling pathway includes *Ehd1* (*Early heading date 1*) and *Ghd7* (*Grain number, plant height and heading date 7*). *Ehd1* encodes a B-type response regulator, which promotes flowering. *Ehd1* affects the levels of *Hd3a* and *RFT1* transcripts [18]. *Ghd7* encodes a CCT (CO, CO-LIKE, and TIMING OF CAB1)-domain protein. *Ghd7* represses *Ehd1*, *Hd3a*, and *RFT1* under LD conditions, but does not affect *Hd1* mRNA levels [19]. Many other genes for heading date have been identified, and their genetic pathways have been well characterized in rice [2,20].

A wide range of variation in heading date has been observed among rice accessions [3,8,21]. More than 650 QTLs for heading date have been detected using segregating populations derived from crosses among rice accessions and wild relatives; they are distributed over all 12 rice chromosomes (Q-TARO database; http://qtaro.abr.affrc.go.jp/ [22]; Gramene QTL database; http://archive.gramene.org/qtl/ [23]). To date, 13 QTLs have been cloned by map-based cloning strategies (OGRO database; http://qtaro.abr.affrc.go.jp/ogro [24]): *Hd1* [10], *Hd6* [12], *Hd3a* [11], *Ehd1* [18], *Ghd7* [19], *DTH8* (*Days to heading on chromosome 8* [25]), *DTH3* (*Days to heading on chromosome 3* [26]), *Hd17* (*Heading date 17* [27]), *DTH2* (*Days to heading on chromosome 2* [28]), *Hd16* (*Heading date 16* [29,30]), *RFT1* [15-17], *Ehd4* (*Early heading date 4* [31]), and *OsPRR37* (*Oryza sativa pseudo-response regulator 37* [32]). Sequence analysis of these genes indicated that allelic differences contribute greatly to heading date variation [9,21]. For example, functional and nonfunctional alleles of *Hd1* are associated with late and early flowering, respectively, and *Hd1* is a major determinant of natural variation in heading date in cultivated rice [10,33]. Deficient or weak alleles of *Ghd7*, *DTH8*, *DTH2*, *Hd16*, and *OsPRR37* are distributed in northern cultivation areas at high latitudes [19,25,28-30,32,34], strongly suggesting that such deficient and weak alleles are involved in the expansion of rice cultivation areas. Favorable alleles were probably selected by breeders to enhance rice productivity and adaptability for each cultivation region.

Genome-wide studies have revealed the divergence of the genetic architecture of flowering time or heading date control in other plant species such as Arabidopsis and maize [35,36]. In Arabidopsis, flowering time variation is controlled by allelic differences of a small number of genes with large genetic effects [36], whereas in maize natural variation of heading date is controlled by the additive effect of many QTLs with small effects [35]. We previously reported a QTL mapping study using 12 F_2_ populations derived from crosses of the *japonica* rice accession Koshihikari (KSH), a common parental line, with diverse accessions originating from various regions of Asia [21]. The study detected one to four QTLs with large effect in each F_2_ population; however, it also indicated that these QTLs cannot fully explain the varietal differences in heading date in some cross combinations. Generally, it is difficult to detect QTLs with small effects in primary mapping populations, e.g., F_2_ populations [37]. Therefore, it is very likely that additional QTLs are also involved in the phenotypic variation for heading date in these populations.

To reveal the genetic architecture of natural variation for heading date in rice by detecting the hidden QTLs, we developed advanced-backcross populations (>14,000 plants) derived from crosses with the same F_2_ populations. Advanced-backcross populations are promising materials for detecting a lot of QTLs involved in variation of heading date in Asian rice accessions. Detection both of large- and small-effect QTLs enable us to estimate the genetic architecture of heading date of Asian rice accessions. We compared genomic positions between detected QTLs and rice heading date genes previously isolated using the map-based cloning strategy, and investigated sequence polymorphisms of the heading date genes in Asian rice accessions. We also discuss similarities and differences in the genetic architectures of heading date (flowering time) among plant species.

## Methods

### Plant materials

We selected 11 rice accessions that originate from various regions of Asia to develop diverse backcrossed populations derived from crosses of these accessions with the *japonica* accession KSH as a common parental and recurrent line (Table 1; Additional file 1: Figure S1). The accessions (5 *indica*, 3 *aus*, and 3 *japonica*) were selected on the basis of their geographical origin, cluster analysis of genome-wide RFLP data, and variation in days to heading (DTH) from a representative rice collection [38,39]. These accessions were used previously as donor parents to produce F_2_ populations [21] and backcrossed inbred lines at BC_1_F_6_ generation [40]. Crosses were performed with F_1_ derived from crosses between those accessions and KSH, and then backcrosses were performed to produce BC_1_F_1_, BC_2_F_1_, BC_3_F_1_, and BC_4_F_1_ individual plants (Additional file 2: Figure S2). From 29 to 39 individual plants were backcrossed in each generation of all of the 11 cross combinations. BC_4_F_1_ plants were self-pollinated to produce BC_4_F_2_ progenies, and BC_4_F_2_ plants were self-pollinated to produce BC_4_F_3_ progenies. We used BC_4_F_2_ populations for QTL detection, and BC_4_F_3_ populations for confirmation of additive effects and chromosomal regions of the putative QTLs.

**Table 1:** **List of 12 diverse accessions in Asian rice and their heading dates**

						**DTH** ^**c**^	
**Accession**	**ID** ^**a**^	**Abbreviation**	**Subspecies**	**Cultivar group** ^**b**^	**Origin**	**ND**	**SD**
Koshihikari		KSH	japonica	A	Japan	106.6 ± 0.6	49.5 ± 0.5
Hayamasari		HAY	japonica	A	Japan	71.8 ± 1.5	54.5 ± 0.7
Qiu Zhao Zong	WRC10	QZZ	indica	C	China	88.2 ± 0.8	62.5 ± 1.0
Tupa 121-3	WRC32	TUP	aus	B	Bangladesh	102.4 ± 1.5	68.0 ± 1.1
Muha	WRC25	MUH	aus	B	India	105.6 ± 1.1	71.2 ± 2.9
Basilanon	WRC44	BAS	aus	B	Philippines	115.0 ± 3.0	116.3 ± 2.5
Deng Pao Zhai	WRC19	DPZ	indica	C	China	119.2 ± 0.5	61.9 ± 3.8
Khau Mac Kho	WRC48	KMK	japonica	A	Vietnam	126.2 ± 0.4	86.7 ± 1.8
Naba	WRC05	NAB	indica	C	India	128.0 ± 1.2	66.7 ± 0.8
Bei Khe	WRC03	BKH	indica	C	Cambodia	130.0 ± 1.7	61.9 ± 3.0
Khao Nam Jen	WRC68	KNJ	japonica	A	Laos	186.3 ± 2.9	59.8 ± 1.4
Bleiyo	WRC63	BLE	indica	C	Thailand	190.8 ± 1.0	40.4 ± 0.5

^a^Accession IDs were selected from the world rice collection (WRC) [38].

^b^Cultivar groups are based on the classification of [38]. Groups A, B, and C correspond to japonica, aus, and indica, respectively.

^c^Days to heading (DTH) were scored under different day-length conditions. ND, the experimental field of National Institute ofAgrobiological Sciences, Tsukuba, Ibaraki, Japan (36°N); SD, short-day length condition (10 h light/14 h dark); LD,long-day length condition (14.5 h light/9.5 h dark). DTH is shown as mean ± standard deviation.

### Scoring of DTH

In each BC_4_F_2_ population, 24 plants were grown in 2010 and 2011 in a paddy field at the National Institute of Agrobiological Sciences (NIAS) in Tsukuba, Japan (36°03′N, 140°11′E). In each BC_4_F_3_ population, 96 and 192 plants were grown in 2012 and 2013 in the same paddy field at the NIAS. Seeds were sown in April, and seedlings were transplanted to the paddy field in May (two rows per BC_4_F_2_ and BC_4_F_3_ population with a distance of 18 cm between plants and 30 cm between rows). The mean day-lengths during the cultivation periods were 13.1 h in April, 14.1 h in May, 14.6 h in June, 14.4 h in July, 13.5 h in August, and 12.4 h in September. Average temperatures during the cultivation periods were 17°C in May, 21°C in June, 24°C in July, 26°C in August, and 22°C in September. Cultivation management followed the standard procedures used at NIAS. DTH in the individual backcrossed plants were scored as the number of days from sowing to the appearance of the first panicle. For the parental accessions, DTH were scored in 24 plants per line and mean values were calculated for each line.

The 12 parental accessions were grown in a controlled-environment cabinet under SD conditions (10 h light/14 h dark, at 28°C for 12 h/24°C for 12 h) and LD conditions (14.5 h light/9.5 h dark, at 28°C for 12 h/24°C for 12 h). The relative humidity was maintained at 60% under a photosynthetic photon flux density of 500 μmol m^−2^ s^−1^ provided by metal halide lamps that covered the spectrum from 300 to 1000 nm. DTH in 10 plants of each accession were scored and mean values were calculated for each accession.

### DNA marker analysis

Total genomic DNA of individual backcrossed plants and parental accessions was extracted from 1–3 cm fresh leaves crushed in 250 μL extraction buffer containing 1 M KCl, 100 mM Tris-HCl (pH 8.0), and 10 mM EDTA. DNA was precipitated with 100 μL 2-propanol, washed with 150 μL 70% ethanol, and dissolved in 30 μL buffer containing 1 mM Tris-HCl pH8.0 and 0.1 mM EDTA, pH 8.0. Simple sequence repeats (SSRs) were used as DNA markers for linkage map construction and QTL detection. SSR markers were selected from those described by previous studies [41,42]. Polymorphism detection procedures for the SSR markers have been described by [21]. Gene-specific markers were used to determine precise genomic positions of 13 heading date genes, *Hd1, Hd6, Hd3a, Ehd1*, *Ghd7*, *DTH8*, *DTH3*, *DTH2*, *Hd17*, *Hd16*, *RFT1*, *OsPRR37*, and *Ehd4* [15,21,26-29,31,34,40,43].

### QTL analysis in advanced-backcross populations

For linkage mapping, version 3.0 of MAPMAKER/EXP [44] was used. The Kosambi mapping function was used to calculate genetic distances [45]. QTL analysis was performed using composite interval mapping as implemented by the Zmapqtl program provided by version 2.5 of the QTL Cartographer software [46]. Genome-wide threshold values (α = 0.05) were used to detect QTLs based on the results of 1,000 permutations. LOD thresholds from 2.0 to 2.8 were used in the QTL analyses of the BC_4_F_3_ populations.

### Sequencing of a heading date gene *DTH8*

All exons of the *DTH8* gene were amplified with specific primers [34] by PCR on genomic DNA of the 12 rice accessions. Amplified DNA fragments were purified and sequenced with the Sanger dideoxy terminator method [47]. To ensure that the sequence data were of high quality (phred score >30), re-sequencing was performed when necessary. Each sequence read was individually mapped onto the Nipponbare reference coding region sequence to ensure that all exons of *DTH8* were covered.

## Results

### Variation in heading date of Asian rice accessions

DTH of the 12 rice accessions varied from 71.8 (extremely early) to 190.8 (extremely late) under natural-day length (ND) conditions (Table 1). Four rice accessions, Hayamasari (HAY), Qiu Zhao Zong (QZZ), Tupa 121-3 (TUP), and Muha (MUH) had earlier heading than KSH. Seven accessions, Basilanon (BAS), Deng Pao Zhai (DPZ), Khao Mac Kho (KMK), Naba (NAB), Bei Khe (BKH), Khao Nam Jen (KNJ), and Bleiyo (BLE), had later heading than KSH. BLE and KNJ showed extremely late heading in comparison with KSH (>80 days). Under SD conditions, BLE, KSH, and HAY had relatively early heading, whereas KMK and BAS had late heading (Table 1; Additional file 1: Figure S1). Under LD conditions, HAY and QZZ had relatively early heading, whereas BAS, KNJ, and BLE had late heading. In BLE, DTH was >280 under LD conditions (Table 1; Additional file 1: Figure S1). DTH of HAY, QZZ, and BAS was similar under ND, SD, and LD conditions, indicating that these accessions are photoperiod-insensitive. DTH of NAB, KNJ, and BLE was much lower under SD conditions than under LD conditions, indicating that these accessions are strongly photoperiod-sensitive.

### Variation in heading date in the BC_4_F_2_ populations

**Figure 1:**
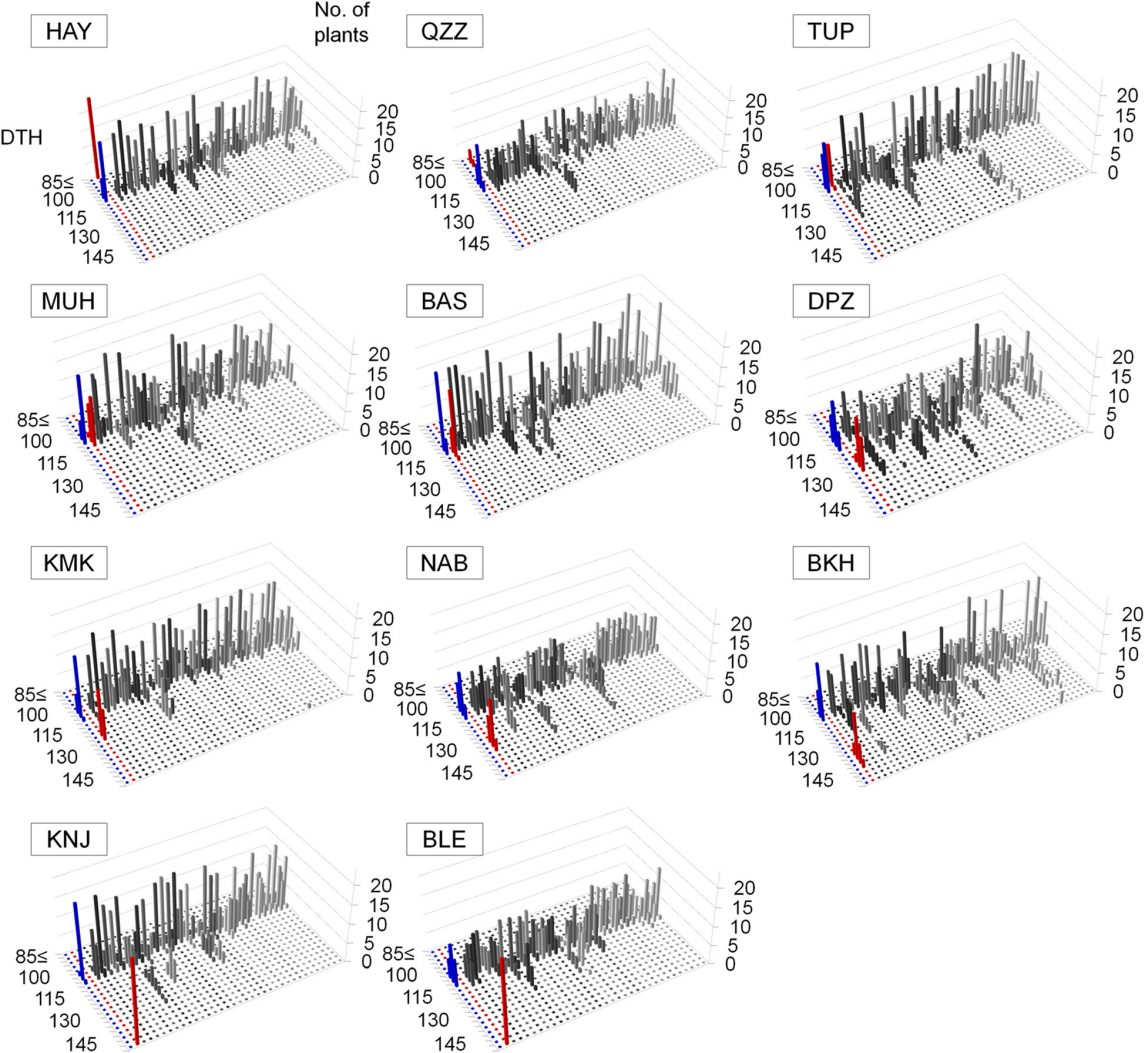
Frequency distributions of days to heading (DTH) under natural day-length conditions in BC_4_F_2_ populations. The 366 BC_4_F_2_ progenies were derived from crosses between Koshihikari (KSH) and 11 diverse accessions of Asian rice. Abbreviations of rice accessions are defined in Table 1. *X*-axis indicates parental accessions and BC_4_F_2_ populations, *Y*-axis indicates DTH, and *Z*-axis indicates the number of individual plants. Bars indicate DTH of KSH (blue), of the other accessions (red) and of backcrossed populations (shaded). DTH was defined as the number of days from sowing to the appearance of the first panicle of individual plants.

We developed BC_4_F_2_ populations in which particular heterozygous chromosome region(s) of donor accessions segregated in the KSH genetic background (Additional file 2: Figure S2; Additional file 3: Figure S3). In each cross combination between KSH and the 11 donor accessions, ~39 BC_4_F_2_ populations were developed (366 BC_4_F_2_ populations, >8,700 backcrossed individual plants in total). These BC_4_F_2_ populations covered the whole genomes of the 11 donor accessions. Most plants in the BC_4_F_2_ populations and the recurrent parent KSH showed similar numbers of DTH (statistically non-significant at the 5% level by the Dunnett’s multiple comparison test). However, several plants showed earlier or later heading than KSH, indicating that heading date QTLs were segregating in the heterozygous chromosomal regions in these BC_4_F_2_ populations. BC_4_F_2_ plants with heading date earlier than that of KSH were observed in all cross combinations except KNJ/KSH and BLE/KSH populations (Figure 1; Additional file 4: Table S1). BC_4_F_2_ plants with later heading than that of KSH were observed in all 11 cross combinations. No BC_4_F_2_ plants had similar heading date with HAY, QZZ, KNJ, or BLE, i.e. the extremely early- or late-heading donor accessions.

### QTL detection in the BC_4_F_2_ populations

**Figure 2:**
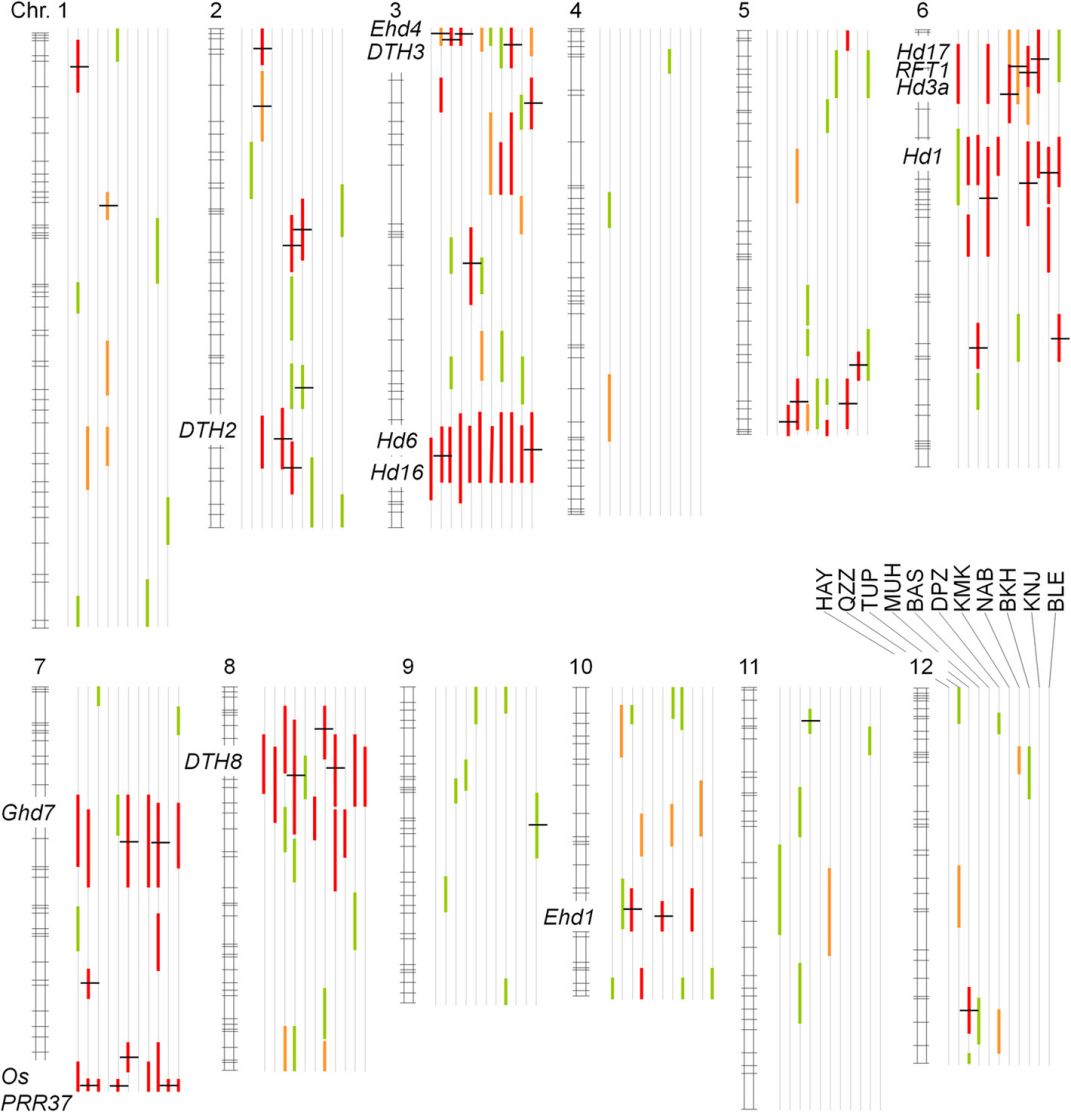
Chromosomal locations of QTLs for days to heading (DTH) under natural day-length conditions detected in BC_4_F_2_ populations. QTLs were detected in 366 BC_4_F_2_ populations derived from crosses between Koshihikari (KSH) and 11 diverse accessions of Asian rice. Consensus linkage maps of 12 rice chromosomes are depicted as ladder-structured boxes; approximate locations of 13 heading date genes isolated previously are shown. QTL positions are oriented from Hayamasari (HAY) (left) to Bleiyo (BLE) (right) in the same order as in Table 1. Vertical bars indicate confidence intervals of QTLs (2-LOD reduction on each side of the peak) and show peak LOD scores of 2.0–3.0 (green), 3.0–4.0 (orange), and >4.0 (red). Horizontal thick bars on the QTL intervals indicate those confirmed in 53 BC_4_F_3_ populations.

In the 366 BC_4_F_2_ populations, a total of 255 QTLs were detected with the LOD scores of >2.0 (Figure 2; Additional file 5: Table S2). Among them, 173 had a LOD score of >3.0 and 134 had a score of >4.0. Previously, 13 heading date QTLs have been isolated and assigned to specific photoperiod flowering pathways in rice [8,9]. Among the 255 newly detected QTLs, 128 corresponded well to genomic positions of the 13 heading date genes (Figure 2; Additional file 5: Table S2). At the position of *Hd1* gene (chromosome 6), 34 QTLs were detected. At the position of *Ghd7* gene (chromosome 7), 10 QTLs were detected. At the position of *DTH8* gene (short arm of chromosome 8), 12 QTLs were detected. Near *Hd17*, *RFT1*, and *Hd3a* genes (short arm of chromosome 6), 13 QTLs were detected. Near *Hd6* and *Hd16* genes (long arm of chromosome 3), 24 QTLs were detected. Near *DTH2* gene (long arm of chromosome 2), 6 QTLs were detected. Near *Ehd4* and *DTH3* genes (short arm of chromosome 3), 14 QTLs were detected. Near *OsPRR37* gene (long arm of chromosome 7), 10 QTLs were detected. And, near *Ehd1* gene (chromosome 10), 3 QTLs were detected. Almost all of these QTLs corresponded well to those detected in F_2_ populations derived from the same cross combinations in the previous study [21].

The remaining 127 of the 255 QTLs were found in genomic regions different from those of the 13 isolated genes (Figure 2; Additional file 5: Table S2). LOD scores >3.0 were detected for 55 QTLs and LOD scores >4.0 were detected for 29 QTLs. These QTLs were distributed over all 12 chromosomes. QTL clusters were found in several chromosomal regions, such as the proximal region of the short arm on chromosome 3, distal end of the long arm on chromosome 5, and centromeric region of chromosome 8 (Figure 2). Our results indicate that, in addition to the alleles of the previously isolated genes, a number of QTLs contribute to phenotypic variation in heading date of Asian rice accessions.

**Figure 3:**
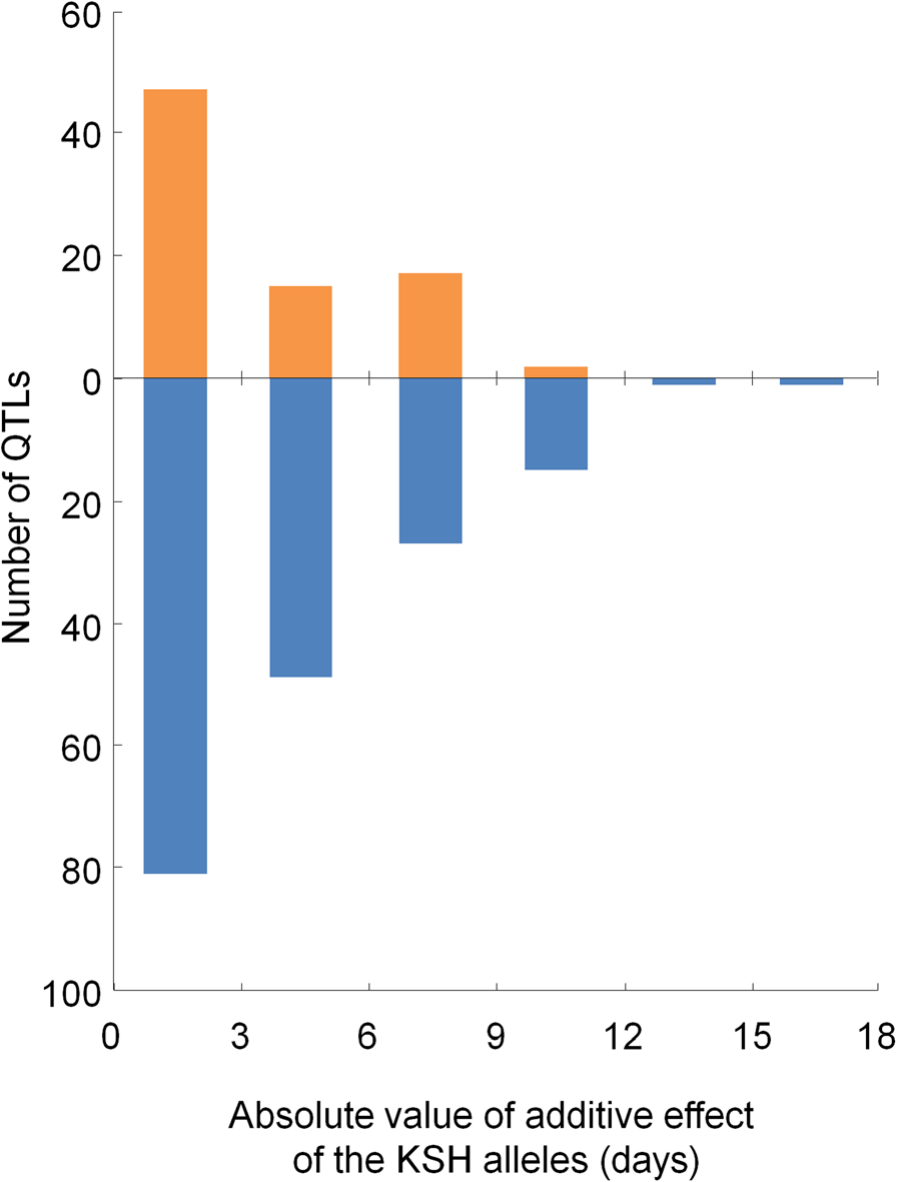
The number of QTLs and their additive effects detected in BC_4_F_2_ populations. The 366 BC_4_F_2_ progenies were derived from crosses between Koshihikari (KSH) and 11 diverse accessions of Asian rice. Orange bars indicate KSH alleles of the QTLs contributing to later flowering in comparison with alleles of other accessions, whereas blue bars indicate KSH alleles of the QTLs contributing to earlier flowering in comparison with alleles of other accessions.

Among the 255 QTLs, the values of significant additive effects of the KSH alleles ranged from −15.1 to 10.9 days (Figure 3; Additional file 5: Table S2) in comparison with the donor parent alleles. In 174 QTLs (68.2%), the KSH alleles showed earliness additive effects, whereas in 81 QTLs (31.8%), the KSH alleles showed lateness additive effects. In 130 QTLs (51.0%), additive effects of KSH alleles of <3 days were observed, whereas in 125 QTLs (49.0%) these effects were >3 days. We detected similar numbers of QTLs showing small or large additive effects in this study. The 128 QTLs located near the 13 genes isolated previously had relatively large additive effects, whereas the other 127 QTLs had relatively small additive effects (Additional file 5: Table S2).

**Figure 4:**
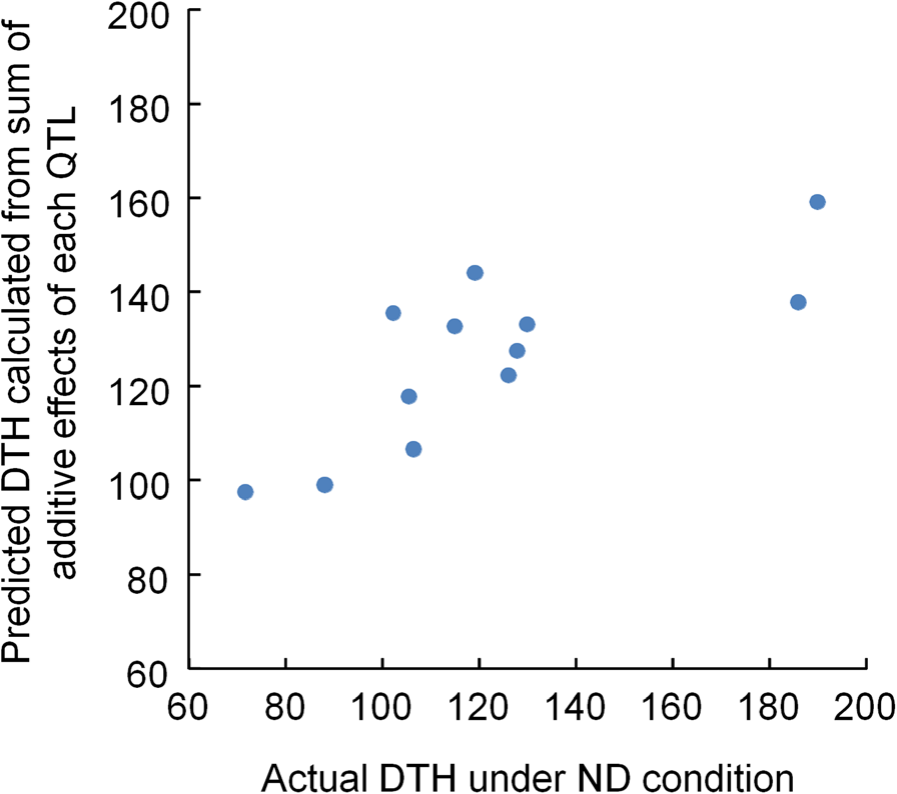
Relationship between actual days to heading (DTH) and predicted DTH estimated from additive effects of each QTL. Actual DTH were scored under natural-day length (ND) condition. Predicted DTH were estimated from the sum of additive effects of each QTL detected in all 366 BC_4_F_2_ populations derived from KSH and 11 diverse accessions of Asian rice.

The cummulative additive effects of QTLs detected in 12 accessions and their DTH under ND conditions showed a significant correlation (*R*^*2*^ = 0.78, Figure 4). Total additive effects of all QTLs reliably predicted the order of heading dates of the 12 donor accessions. HAY and QZZ were predicted to have early heading dates, whereas KNJ and BLE were predicted to have late heading. The predicted heading dates had the same order as the actual heading dates under ND condition in the 12 rice accessions. However, the predicted heading dates deviated from actual heading dates under ND conditions in extremely-early and extremely-late heading accessions. Actual heading dates of HAY and QZZ were 34.8 and 18.4 days earlier, respectively, than that of KSH under ND conditions (Table 1), whereas predicted heading dates of HAY and QZZ were only 9.1 and 7.6 days earlier. Actual heading dates of KNJ and BLE were 79.4 and 83.4 days later, respectively, than that of KSH under ND conditions (Table 1), whereas predicted heading dates were 31.2 and 52.5 days later.

### Confirmation of QTLs in the BC_4_F_3_ populations

In small-sized populations, it may be difficult to detect reliable QTLs because of the possibility of false positive detection [37]. To confirm the genetic effects of the QTLs detected in the BC_4_F_2_ populations, we selected 56 QTLs that included both large- and small-effect QTLs distributed across the rice genome. We developed and analyzed 53 BC_4_F_3_ populations consisting of >6,000 backcrossed individual plants (96 or 192 plants in each population). In these BC_4_F_3_ populations, we confirmed the presence of all 56 QTLs detected in the BC_4_F_2_ populations (Table 2; Additional file 5: Table S2).

**Table 2:** Heading date QTLs confirmed in BC_4_F_3_ populations

**Population**	**Chromosome**	**Physical position ofQTL (Mbp)**	**Marker interval**	**LOD** ^**a**^	**Additive effect** ^**b**^	**Dominance effect** ^**c**^	**PVE (%)** ^**d**^	**Corresponding gene** ^**e**^	**Located near gene** ^**f**^
QZZ	1	0.2–10.8	RM6887–RM1287	5.3	1.7	0.2	20.0		
	3	0.5–5.5	RM4108–RM5442	12.1	-1.7	0.2	52.1		Ehd4, DTH3
	3	30.4–35.6	RM3199–RM3329	36.2	-8.1	4.0	77.5	Hd6	Hd16
	7	7.1–13.4	RM21251–RM7273	32.1	10.9	8.6	89.9	Ghd7	
	7	26.8–29.4	RM1364–RM22164	2.4	3.3	-0.1	5.7		OsPRR37
TUP	2	1.9	RM7562	8.3	1.6	0.0	37.6		
	2	6.7–11.3	RM5897–RM1234	6.8	-1.4	-0.2	26.4		
	2	27.1–30.6	RM1367–RM3316	4.1	-0.5	-0.8	13.2		DTH2
	3	0.5	RM4108	19.0	-2.4	-0.6	54.6		Ehd4, DTH3
	4	2.0–11.6	RM5414–RM16606	2.8	0.7	-0.8	20.9		
	5	23.9–29.5	RM3476–RM3286	14.6	-1.3	0.4	35.0		
	6	24.5–26.0	RM5957–RM6395	5.0	-0.8	-0.1	22.5		
	10	11.7–17.4	RM4455–RM5620	2.5	-0.5	-0.9	18.3		Ehd1
	12	20.0–24.4	RM28305–RM5479	8.5	-0.9	-0.4	19.7		
MUH	3	0.5–1.5	RM4108–RM3372	12.6	-2.0	0.3	47.6		Ehd4, DTH3
	5	23.9–27.9	RM3476–RM5784	9.5	-1.9	0.4	16.1		
	6	8.1–8.8	RM19725–RM5963	26.1	4.2	-1.1	67.3	Hd1	
	6	15.8–20.3	RM20023–RM7193	20.8	4.6	-0.4	66.1		
	8	6.8–10.3	RM22617–RM3395	25.0	-4.0	0.5	74.0	DTH8	
	11	3.8–8.1	RM5599–RM3701	2.2	0.5	-0.9	3.9		
BAS	2	11.3–13.5	RM1234–RM13106	4.4	-0.6	0.0	9.8		
	2	33.0–35.4	RM7286–RM3850	3.6	-1.1	0.7	8.5		DTH2
	3	6.9–10.1	RM3872–RM14778	7.0	-1.1	0.2	17.4		Ehd4, DTH3
	3	10.1–14.5	RM14778–RM6959	4.3	-1.1	0.7	9.8		
	3	17.4–21.4	RM1334–RM5488	7.9	-0.9	-0.4	16.7		
	7	29.4	RM22164	21.0	-3.7	-0.1	69.7		OsPRR37
DPZ	2	13.4–18.4	RM13101–RM1211	2.5	-1.0	0.0	5.4		
	2	29.3–35.4	RM6933–RM3850	9.2	-1.1	0.3	38.3		DTH2
	6	0.2	RM6467	39.5	-3.6	-3.3	85.2		
	7	13.2–16.2	RM21433–RM5481	10.6	-4.0	-0.9	42.5	Ghd7	
	7	28.1–29.4	RM22105–RM22164	31.6	-5.0	0.0	53.4		OsPRR37
	10	17.5–20.7	RM5620–RM25771	5.3	0.5	0.1	18.2		Ehd1
	12	7.1–10.1	RM27792–RM6973	5.7	-0.6	-0.1	20.1		
KMK	2	4.3–9.5	RM4355–RM12921	2.2	0.5	-0.3	7.8		
	2	20.1–24.0	RM1379–RM3515	2.1	0.5	0.2	7.3		
	6	8.1	RM19725	34.4	4.0	-0.8	81.0	Hd1	
	8	3.0–3.7	RM4955–RM1148	9.5	-1.3	0.2	45.4		
NAB	2	27.1–34.7	RM1367–RM3789	2.2	-0.6	0.1	5.5		DTH2
	3	9.9–15.0	RM1371–RM3204	13.4	-1.6	0.2	43.6		Ehd4, DTH3
	5	27.9–29.7	RM5784–RM19218	7.0	-1.0	0.2	18.7		
	6	0.2–2.2	RM6467–RM8112	10.7	1.3	0.1	29.1		Hd17
	6	8.8–13.0	RM5963–RM19951	30.2	5.9	0.6	79.2	Hd1	
	8	5.9	RM6838	5.5	-1.8	0.3	7.9	DTH8	
	9	14.4–16.7	RM5657–RM6235	2.9	0.5	-0.1	4.5		
BKH	3	0.5–5.5	RM4108–RM5442	13.9	-3.8	-0.5	46.2		Ehd4, DTH3
	5	22.3–27.9	RM3295–RM5784	7.1	-1.3	-0.2	13.3		
	6	0.2–5.2	RM6467–RM5754	18.3	-2.5	0.1	59.6	RFT1, Hd3a	Hd17
	7	13.4–18.4	RM7273–RM6394	16.9	-2.2	0.3	60.8	Ghd7	
	8	10.3–14.7	RM3395–RM22896	9.4	3.4	0.6	36.6	DTH8	
KNJ	5	23.9–27.9	RM3476–RM5784	5.7	-1.8	-0.2	25.7		
	6	16.1–29.8	RM3615–RM20045	15.5	-4.6	1.1	31.3		
	7	26.8–29.4	RM1364–RM22164	18.3	-2.0	0.5	63.3		OsPRR37
	9	3.4–9.2	RM23736–RM1328	2.7	-1.0	0.3	4.4		
BLE	3	0.5	RM4108	20.1	-2.8	-0.5	65.9		Ehd4, DTH3
	3	30.4–35.6	RM3199–RM3329	32.1	-5.2	3.4	81.2	Hd6	Hd16
	6	26.0–28.5	RM6395–RM1370	3.4	-1.4	-0.3	6.9		

^a^Log-likelihood value. LOD threshold to detect QTLs was determined in each BC_4_F_3_ population.

^b^Additive effect of KSH allele on days to heading.

^c^Dominance effect of KSH allele on days to heading.

^d^Percentage of phenotypic variance explained by QTL.

^e^Previously identified heading date genes corresponding to the QTLs detected in this study based on their physical positions on IRGSP 1.0.

^f^Previously identified heading date genes located near the QTLs detected in this study based on their physical positions on IRGSP 1.0.

The BC_4_F_3_ populations were derived from crosses between Koshihikari (KSH) and 11 diverse accessions of Asian rice. Abbreviations of rice accessions are described in Table 1.

**Figure 5:**
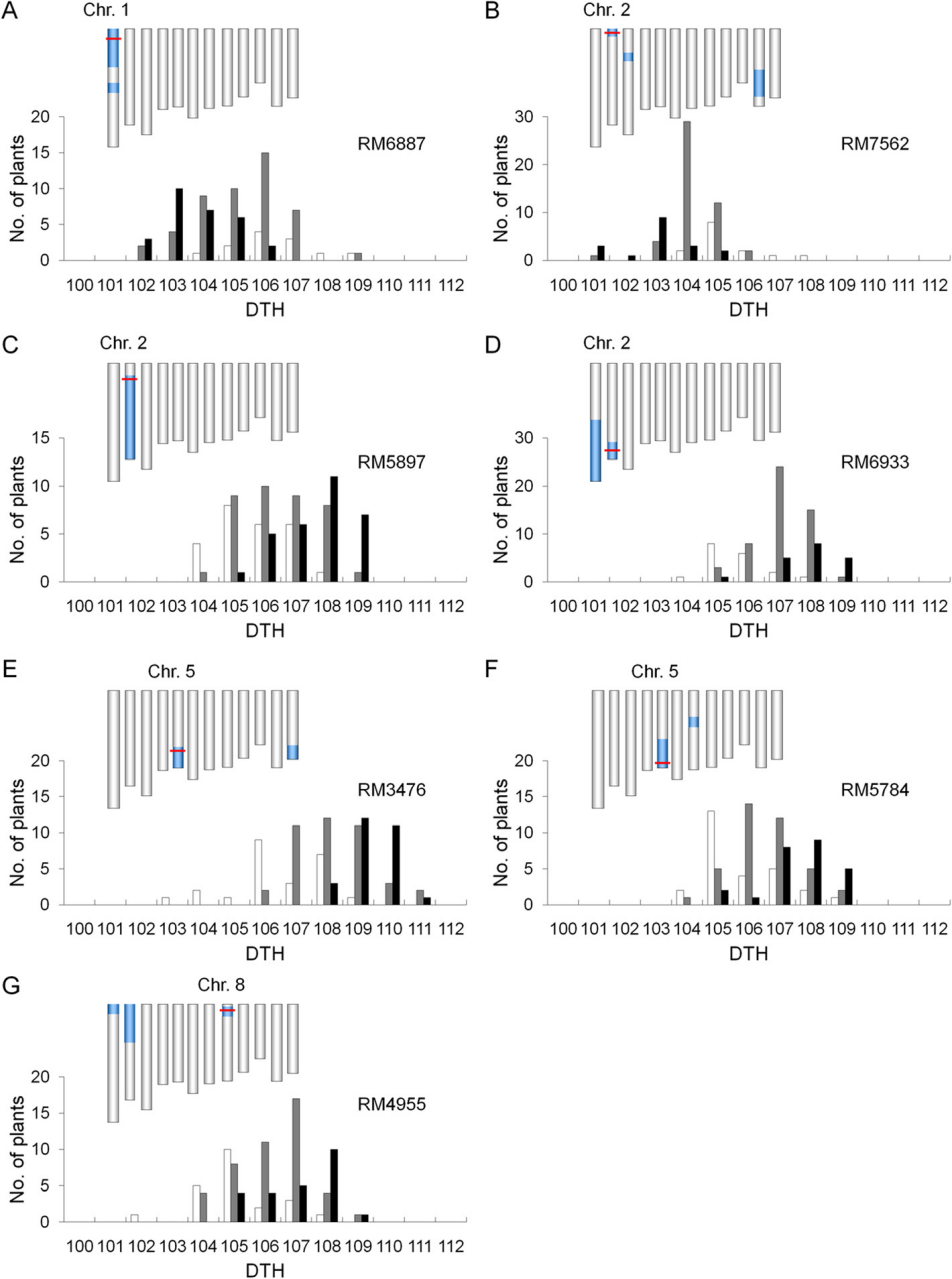
Confirmation of the allelic differences at seven QTLs using BC_4_F_3_ populations. In each panel, graphical representation of the genotype of a BC_4_F_2_ plant is shown in the upper part and frequency distribution of days to heading (DTH) in seven BC_4_F_3_ populations is shown in the lower part. In KSH/QZZ **(A)**, KSH/TUP **(B,C)**, KSH/DPZ **(D)**, KSH/TUP **(E)**, KSH/KNJ **(F)** and KSH/KMK **(G)** populations. In genotypes, vertical bars indicate genotypes of rice chromosomes from 1 (left) to 12 (right). Bars indicate genotypes heterozygous in blue, and homozygous for KSH alleles in white. QTL positions detected in BC_4_F_2_ and BC_4_F_3_ populations are depicted as red horizontal lines. In the lower part of each panel, bars correspond to the nearest molecular markers homozygous for KSH allele (white), heterozygous (gray), and homozygous for the allele from another accession (black).

Among small-effect QTLs, we focused on seven QTLs chosen on the basis of the size of additive effect and genomic position (Figure 5): these QTLs had additive effects of <3 days and their locations were different from those of heading date genes isolated previously. In QZZ/KSH, the additive effects of the KSH alleles of the QTL on the short arm of chromosome 1 were 2.2 days in the BC_4_F_2_ population and 1.7 days in the BC_4_F_3_ population. In TUP/KSH, the additive effects of the KSH alleles of the two QTLs on the short arm of chromosome 2 were 2.1 and −1.6 days in the BC_4_F_2_ populations and 1.6 and −1.4 days in the BC_4_F_3_ populations. In DPZ/KSH, the additive effects of the KSH alleles of the QTL on the long arm of chromosome 2 were −1.9 days in the BC_4_F_2_ population and −1.1 days in the BC_4_F_3_ population. In TUP/KSH, the additive effects of the KSH alleles of the QTL on the long arm of chromosome 5 were −1.1 days in the BC_4_F_2_ population and −1.3 days in the BC_4_F_3_ population. In KNJ/KSH, the additive effects of the KSH alleles of the other QTL on the long arm of chromosome 5 were −2.2 days in the BC_4_F_2_ population and −1.8 days in the BC_4_F_3_ population. In KMK/KSH, the additive effects of the KSH alleles of the two QTLs on the short arm of chromosome 8 were −2.5 days and −1.3 days in the two BC_4_F_2_ populations, and −1.3 days in the BC_4_F_3_ populations. Using the BC_4_F_3_ populations, we even confirmed the existence of small-effect QTLs with additive effects of <3 days.

**Figure 6:**
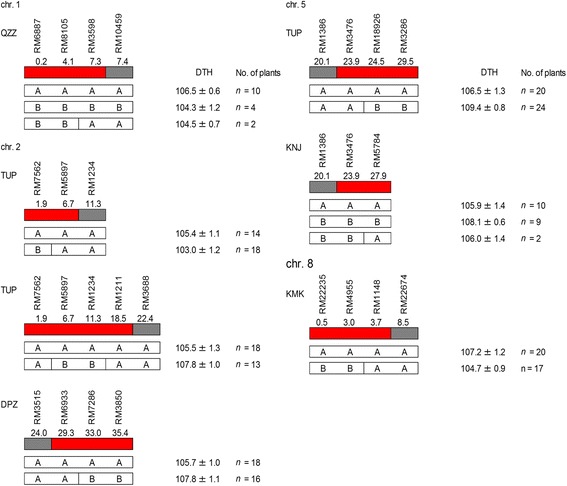
Fine mapping of seven QTLs for days to heading (DTH) under natural day-length conditions in BC_4_F_3_ populations. BC_4_F_3_ progenies were derived from crosses between Koshihikari (KSH alleles[marked with A]) and other accessions of Asian rice (QZZ, TUP, DPZ, and KNJ alleles [marked with B]). Values are means ± standard deviation. Abbreviations of rice accessions are defined in Table 1. Positions of molecular markers are indicated according to IRGSP 1.0 of the rice genome sequence [52,53]. Red bars indicate marker intervals delimited for each QTL position.

For the same seven QTLs, we tried to delimit their chromosomal regions by substitution mapping in the BC_4_F_3_ populations, even though only a small number of BC_4_F_3_ progenies had recombination within the QTL regions (Figure 6). In the QZZ/KSH population, the QTL on the short arm of chromosome 1 was located within ~7.3 Mbp of the marker interval from the distal end of the arm to RM3598. In the TUP/KSH population, the two QTLs on the short arm of chromosome 2 were located within ~6.7 Mbp of the interval from the distal end of the arm to RM5897 and within ~16.6 Mbp of the RM7562–RM1211 interval. In the DPZ/KSH population, the QTL on the long arm of chromosome 2 was located within ~6.1 Mbp of the interval from the distal end of the arm to RM6933. On the long arm of chromosome 5, the two QTLs were narrowed down to within ~5.6 Mbp of the interval from the distal end of the arm to RM3476 in the TUP/KSH population, and within ~4.0 Mbp of the interval from the distal end of the arm to RM3476 in the KNJ/KSH population. In the KMK/KSH population, the QTL on the short arm of chromosome 8 was located within ~3.7 Mbp of the interval from the distal end of the arm to RM1148. Our results clearly delimit significant marker intervals that include small-effect QTLs using advanced-backcross progenies.

## Discussion

Many genetic studies have focused on cloning genes or QTLs for heading date in rice, and a detailed genetic control pathway has been revealed [2,8,9]. In most cases, QTLs with large effects have been studied as targets for genetic analysis and molecular characterization. The role of QTLs with small-effects in heading date variation among rice accessions still remains unclear.

We demonstrated the potential utility of advanced-backcross populations in genetic analysis of natural variation in heading date, in particular, detection of small-effect QTLs. We found that 130 QTLs (51.0%) had additive effects of <3 days. Most of these QTLs were found in different chromosomal regions than the previously isolated genes. Previous study [48] detected heading date QTLs in the F_2_ populations (4 QTLs) and advanced-backcross populations (12 QTLs) derived from crosses between KSH and the *indica* accession Nona Bokra. The QTLs detected in the advanced-backcross populations showed smaller additive effects than the QTLs detected in the F_2_ populations. Therefore, the results of the previous and current studies clearly indicate that advanced-backcross populations are more efficient for detection of small-effect QTLs than the F_2_ populations.

Small-effect QTLs often show inconsistent additive effects across different genetic backgrounds and environmental conditions. However, in this study, a number of small effect QTLs were consistently detected both in the BC_4_F_2_ and BC_4_F_3_ populations. In the 366 BC_4_F_2_ populations, QTLs with LOD >2.0 (255 QTLs), including those with LOD >3.0 (173 QTLs) and >4.0 (134 QTLs) were detected. Among the 255 QTLs, we selected 56 QTLs and confirmed their presence in the BC_4_F_3_ populations. These results also indicate that advanced-backcross populations are suitable for detection of small-effect QTLs even in small-sized BC_4_F_2_ populations used in this study.

The advanced-backcross populations are also suitable to delimit the positions of individual small-effect QTLs. For example, in the TUP/KSH populations, two QTLs with opposite genetic effects were detected in a very narrow region on the short arm of chromosome 2. However, these QTLs are independent of each other because of the opposite additive effects of KSH alleles. One QTL was localized closer to the distal end of the short arm of chromosome 2 than the other QTL, which was located within the marker interval from RM7562 to RM1211. In KMK/KSH populations, one QTL was detected in the region from the distal end to RM1148 (3.7 Mbp) on the short arm of chromosome 8. This QTL was located close to *DTH8* gene. However, *DTH8* gene is located at 4.3 Mbp from the distal end of the short arm of chromosome 8, and KMK and KSH share the same allele of *DTH8* gene (Additional file 6: Figure S4). Therefore, this QTL and *DTH8* gene are clearly distinct from each other. These examples clearly demonstrate that substitution mapping using advanced-backcross populations is effective for dissecting two independent QTLs located closely to each other.

Previous QTL studies of heading date have identified several small-effect QTLs (*Hd4*, *Hd7*, *Hd8*, *Hd9*, *Hd10*, *Hd12*, *Hd13*, and *Hd17* [27,49-51]). These small-effect QTLs remain poorly characterized and further genetic analyses such as gene cloning and functional characterization are necessary to understand the whole genetic architecture controlling heading date in rice. However, map-based cloning of small-effect QTLs is sometimes difficult because of small phenotypic differences between the QTL alleles. Reverse genetic approaches can be applied to solve this problem. Recently, using next-generation sequencing technology, whole genomic sequences of many accessions have been obtained. Sequence comparison of a particular QTL region among rice accessions is a practical way to nominate probable candidate genes with functional polymorphisms, such as single amino acid substitutions and in-frame deletions or insertions. We can also nominate target genes of interest according to estimation of the gene function based on gene annotation information from Rice Annotation Project Database (http://rapdb.dna.affrc.go.jp/ [52,53]) and comprehensive gene expression profiles of microarray analysis from Rice Expression Profile database (http://ricexpro.dna.affrc.go.jp/ [54]). Once a candidate gene is nominated, we should develop transgenic plants including over-expression lines and RNAi lines can be developed. We can also find lines with disrupted target genes in large sets of mutants with insertions of *Tos17* [55] and T-DNA [56-58]. Recently developed TILLING technique allows direct identification of mutation sites within a specific gene [59-61]. The TILLING technique could provide a powerful strategy to obtain lines with target genes disrupted in mutants generated by irradiation or chemical treatment. The approaches described above can be applied to identify genes for heading date QTLs with quite small effects, such as those detected in this study. To date, in addition to 13 genes isolated by map-based cloning strategies, 60 genes have been identified as flowering-time–related genes by genetic analysis of rice mutants and transgenic lines (OGRO database [24]). In this study, some small-effect QTLs were located near the genes reported in previous studies. Spontaneous mutations in these genes might cause functional polymorphisms of these QTLs. Sequencing analyses of these genes and adjacent regions may reveal the genes responsible for heading date differences among Asian rice accessions.

In this study, the chromosomal positions of several large-effect QTLs corresponded to the positions of *Hd6*, *Hd3a*/*RFT1*, *Hd1*, *Ghd7*, *OsPRR37*, and *DTH8*. Sequence polymorphisms in the isolated genes have been detected in the 12 accessions used in this study (*Hd1* and *Hd3a* [33]; *Hd6* [43]; *Ghd7* [21]; *RFT1* [15]; *DTH8* [Additional file 6: Figure S4]) (Additional file 7: Table S3). Sequence polymorphisms in the isolated genes were consistent with locations of the QTLs in this study. For example, in the HAY/KSH and QZZ/KSH populations, QTLs were found in the same genomic regions as *Hd1*, *Ghd7*, *OsPRR37*, and *DTH8*. HAY and QZZ had alleles of these genes different from that of KSH. In comparison with KSH, HAY had a strong functional allele of *Hd1*, whereas QZZ had a non-functional allele of *Hd1*. HAY and QZZ have non-functional alleles of *Ghd7*, *OsPRR37*, and *DTH8*. The *japonica* cultivars harboring non-functional alleles of both *Ghd7* and *OsPRR37* flower extremely early under ND conditions, and are adapted to the northernmost regions (up to 53°N latitude) of rice cultivation areas in China, Korea, and Japan [34,62]. We also investigated functional nucleotide polymorphisms in *Ehd1*, *Hd17*, *Hd16*, *DTH2*, *Ehd3*, and *Ehd4* (Additional file 7: Table S3). In the HAY/KSH and QZZ/KSH populations, additional QTLs were detected in the *Hd6*/*Hd16*, *Ehd4*/*DTH3*, and *Hd17*/*RFT1*/*Hd3a* regions. Because HAY and KSH have the same allele of *Hd6* and different alleles of *Hd16*, the QTL near *Hd6* and *Hd16* would be due to difference between HAY (functional *Hd16* allele) and KSH (non-functional *Hd16* allele). QZZ and KSH have different alleles of *Hd6* and *Hd16*, but the same alleles of *Ehd4*, *DTH3*, *Hd17*, *RFT1* and *Hd3a*. Therefore, the QTLs near *Hd6* and *Hd16* would be due to allelic differences in these genes, but the QTLs near *Ehd4*, *DTH3*, *Hd17*, *RFT1*, and *Hd3a* are probably due to unidentified genes. These results suggest that heading date variations in rice accessions are due to combinations of different alleles of previously isolated and unidentified heading date genes.

**Figure 7:**
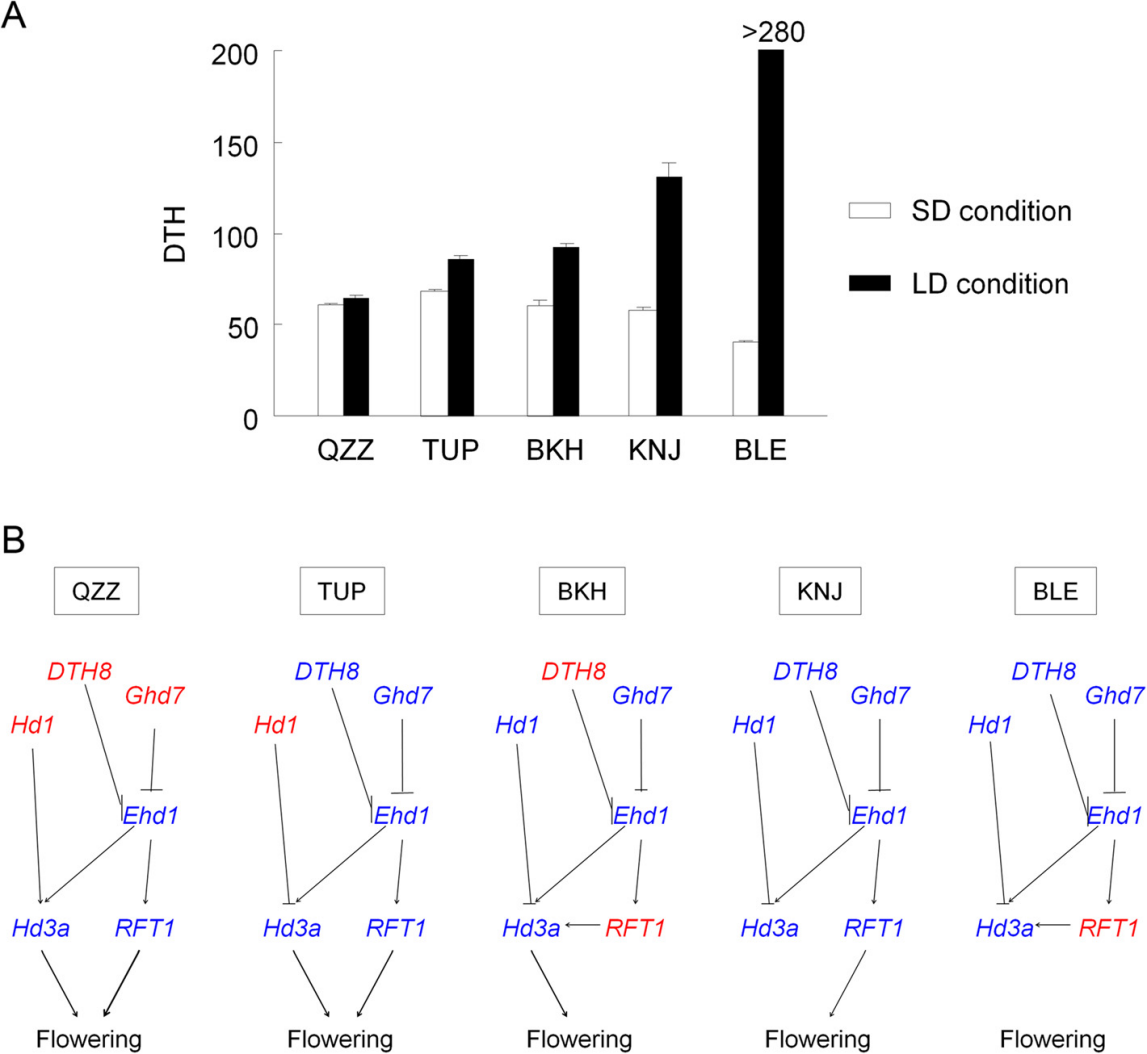
A model of genetic control of responses to day length in different Asian rice. Six genes were included in the model for five representative Asian rice accessions. Abbreviations of rice accessions are defined in Table 1. **(A)** Days to heading (DTH) were scored under short-day length (SD) and long-day length (LD) conditions. SD conditions were 10 h light/14 h dark; LD conditions were 14.5 h light/9.5 h dark. Values are means ± standard deviation (*n* = 10). **(B)** Genetic regulatory pathways under LD conditions. Functional alleles are shown in blue and non-functional alleles are shown in red.

Based on the results in this study, we estimated the relationship between the degree of photoperiod sensitivity and the gene pathway regulating heading date in representative accessions (Figure 7). As QZZ is photoperiod-insensitive due to non-functional alleles of *Hd1*, *Ghd7*, and *DTH8*, *Hd3*a and *RFT1* are transcriptionally up-regulated even under LD conditions. TUP shows weak photoperiod sensitivity due to a non-functional allele of *Hd1*, but the functional allele of *DTH8* would transcriptionally down-regulate *Ehd1* and *RFT1*; as a result, TUP shows a later heading date than QZZ under LD conditions. BKH shows weak photoperiod sensitivity mainly due to non-functional alleles of *DTH8* and *RFT1*. The latter fails to induce heading under LD conditions; as a result, BKH shows a later heading date than QZZ. KNJ has functional alleles of almost all heading date genes and therefore shows strong photoperiod sensitivity. In BLE, extremely late heading date (>280 days) under LD conditions was due to a non-functional allele of *RFT1*. Thus, different allele combinations of the heading date genes in rice accessions can explain the degree of photoperiod sensitivity and heading date variation under various day-length conditions.

Our results suggest that the genetic architecture of heading date in rice is a combination of large-effect QTLs and small-effect QTLs. The sum of additive effects of each QTL reliably estimated the heading date of individual donor accessions (*R*^*2*^ = 0.78), although the prediction deviated from the actual heading date in several accessions. Previous studies have reported a complex regulation system that includes epistatic interactions among genes and QTLs for heading date of rice [2,5,8]. Consideration of epistatic interactions could more accurately predict heading date of each rice accession on the basis of allelic combinations among a number of genes and QTLs. The complete genetic architecture of heading date would enable precise understanding of differences in heading dates of rice accessions and help to develop new rice accessions.

Genome-wide genetic studies have revealed the divergence of the genetic architecture of flowering time or heading date control in *Arabidopsis*, maize, sorghum and barley [35,36,63,64]. In *Arabidopsis*, the genetic basis of flowering time variation is shaped by a small number of genes with large effects such as transcription factor genes *FRIGIDA* (*FRI*) and *FLOWERING LOCUS C* (*FLC*) that act in the vernalization-responsive pathway [36]. The authors found that most heading date QTLs cluster in as few as five genomic regions, which include *FRI* and *FLC*. These results were obtained using 17 F_2_ populations derived from crosses among 18 distinct accessions representing much of the common genetic diversity of *Arabidopsis*. In barley, 17 double haploid populations reveal a set of QTLs, such as *Photoperiod-H1* (*Ppd-H1*), *Ppd-H2*, *Vernalization-H1* (*Vrn-H1*), *Vrn-H2* and *Early maturity 6*, accounted for an important percentage of the heading date variation [64]. It is suggesting that the genetic architectures of heading date are similar between *Arabidopsis* and barley. In maize, large-scale genetic analysis of >5,000 recombinant inbred lines from crosses among 25 diverse inbred lines indicated that heading date is controlled by the additive effect of many QTLs with small effects [35]. In sorghum, 24 populations of recombinant inbred lines of >1,300 individuals revealed that a relatively large number of QTLs with small effects control heading date, and 75% of detected QTLs were localized in the same chromosomal regions as in maize [63]. Large-effect QTLs have been also reported in sorghum; for example, *Ma1* is a major photoperiod sensitivity locus with an additive effect of 40.3 days [65]. Therefore, the genetic architecture of heading date in sorghum is similar to that in rice.

A comparison of the genetic architecture of flowering time or heading date among *Arabidopsis*, barley, maize, and rice should consider three major points. First, even though the cues involved in genetic control of flowering (*Arabidopsis*) and heading (rice and barley), such as temperature and day length, are different, the genetic control is similar; a combination of loss- or gain-of-function alleles of major genes is responsible for large variation in flowering time and heading date. Second, difference in the regulatory mechanisms between rice and *Arabidopsis* (barley) might be due to difference in cultivation areas and reproduction methods of these species. *Arabidopsis* and barley grow at higher latitudes of the Northern hemisphere than rice and maize, and some *Arabidopsis* and barley accessions require vernalization to avoid flowering during prolonged cold conditions in winter [66,67]. Therefore, allelic differences between vernalization-related genes, such as *FRI* and *FLC* in *Arabidopsis*, and *Vrn-H1* and *Vrn-H2* in barley, might contribute considerably to natural variation of flowering time and heading date. Third, the genetic architecture is different in rice and maize and regulation by large-effect QTLs has not been detected in maize. Maize was domesticated in tropical areas at low latitudes and cultivation was expanded to higher-latitude areas during selection by humans [68]. Rice domestication was similar to that of maize [69,70]. However, maize is an out-crossing species, whereas rice is a self-pollinating species. Therefore, in maize, heading date of individual plants must be substantially synchronous within a local population to ensure mating success [35] and selection in maize breeding may have favored accumulation of many small-effect QTLs to ensure adaptation to different environmental conditions.

## Conclusions

Our results enhance understanding of the genetic architecture of heading date in rice. Comparisons of results between the previous and our studies suggested that genetic architecture in rice were different from those in *Arabidopsis*, maize, barley and sorghum. In rice, small-effect QTLs largely contributed to a wide range of natural variations of heading date, in addition to large-effect QTLs including 13 genes isolated previously. Both large- and small-effect QTLs could be of great significance in rice breeding for fine-tuning of heading date, which is needed for adaption to certain environmental conditions (i.e., wide ranges of temperature and day-length) and to expand rice growing areas to high-latitude regions.

## Additional files

Additional file 1: Figure S1. Days to heading (DTH) of Koshihikari (KSH) and 11 diverse accessions of Asian rice under short-day length (SD) and long-day length (LD) conditions. Values are means ± standard deviation (*n* = 10). SD conditions were 10 h light/14 h dark; LD conditions were 14.5 h light/9.5 h dark. Abbreviations of rice accessions are defined in Table 1. Additional file 2: Figure S2. Schematic flow chart of development of advanced-backcross populations at BC_4_F_2_ and BC_4_F_3_ generations. Additional file 3: Figure S3. Graphical representations of genotypes of 366 BC_4_F_2_ populations derived from crosses between Koshihikari (KSH) and 11 diverse accessions of Asian rice. Abbreviations of rice accessions are defined in Table 1. Each horizontal bar corresponds to a rice chromosome; chromosomes are arranged from 1 (left) to 12 (right) with each cell indicating one chromosome of a single population. Each horizontal row indicates the genotype of a BC_4_F_2_ population. Regions heterozygous are shown in black, those homozygous for KSH alleles are shown in white, and those missing alleles are shown in gray. Graphical genotypes are based on physical distances according to IRGSP 1.0 of the rice genome sequence [52,53]. Additional file 4: Table S1. Frequency distributions for days to heading (DTH) in 366 BC_4_F_2_ populations derived from crosses between Koshihikari (KSH) and 11 diverse accessions of Asian rice. Fill in cells show the number of individual plants: less than five (right pink) and more than six (dark pink). Abbreviations of rice accessions are defined in Table 1. Additional file 5: Table S2. List of heading date QTLs detected in BC_4_F_2_ populations derived from crosses between Koshihikari (KSH) and 11 diverse accessions of Asian rice. QTLs written by bold characters indicate those confirmed in BC_4_F_3_ populations. Abbreviations of rice accessions are defined in Table 1. Additional file 6Figure S4. Substitution (boxes) and insertion/deletion (*-*) polymorphisms of amino acids in the DTH8 protein in 12 diverse accessions of Asian rice. Abbreviations of rice accessions are defined in Table 1. The conserved histon-fold motif domain in DTH8 is indicated in orange. Numbers under the DTH8 diagram indicate the positions of polymorphic sites. Numbers on the right side show the total length of each predicted amino acid sequence. The regions with amino acid changes due to a frame shift are labeled with asterisks. Stop indicates a stop codon. Accession numbers of each sequence are DDBJ: LC016712-LC016721. Additional file 7: Table S3. Allele types of 13 heading date genes isolated by previous studies in 12 diverse accessions of Asian rice. Abbreviations of rice accessions are defined in Table 1. Red and blue characters indicate early heading and late heading alleles, respectively. Numbers, nucleotides, and lower-case letters are respective alleles identified in the previous studies: *DTH2* [28]: *Ehd4* [31]: *DTH3* [26]: *Hd6* [43]: *Hd16* [29]: *Hd17* [27]: *RFT1* [15]: *Hd3a* and *Hd1* [33]: *Ghd7* [21]: *OsPRR37* [32]: *DTH8* [25]: *Ehd1* [18]. Complete genomic sequences of *Hd1*, *Hd6*, *Ghd7*, *Hd3a*, *RFT1* and *DTH8* were determined in the 12 diverse accessions. Functional nucleotide polymorphisms of *Ehd1*, *DTH2*, *DTH3*, *Ehd4*, *Hd17*, *Hd16* and *OsPRR37* were genotyped by gene-specific markers in the 12 diverse accessions.

## Abbreviations

QTLs: Quantitative trait loci
DTH: Days to heading
SD: Short-day length
LD: Long-day length
ND: Natural-day length
KSH: Koshihikari
HAY: Hayamasari
QZZ: Qiu Zhao Zong
TUP: Tupa 121-3
MUH: Muha
BAS: Basilanon
DPZ: Deng Pao Zhai
KMK: Khao Mac Kho
NAB: Naba
BKH: Bei Khe
KNJ: Khao Nam Jen
BLE: Bleiyo
OsGI: Rice GIGANTEA
Hd1: Heading date 1
Hd3a: Heading date 3a
Hd6: Heading date 6
RFT1: Rice flowering locus T 1
Ehd1: Early heading date 1
Ghd7: Grain number, plant height and heading date 7
DTH8: Days to heading on chromosome 8
DTH3: Days to heading on chromosome 3
Hd17: Heading date 17
DTH2: Days to heading on chromosome 2
Hd16: Heading date 16
Ehd4: Early heading date 4
OsPRR37: Oryza sativa pseudo-response regulator 37
