# Supplementary material for: Genetic architecture of variation in heading date among Asian rice accessions
Source: BMC Plant Biol. 2015 May 8;15:115. doi: 10.1186/s12870-015-0501-x (PMC4424449; doi:10.1186/s12870-015-0501-x)
Supplement: Additional file 1: Figure S1. — Days to heading (DTH) of Koshihikari (KSH) and 11 diverse accessions of Asian rice under short-day length (SD) and long-day length (LD) conditions. Values are means ± standard deviation (n = 10). SD conditions were 10 h light/14 h dark; LD conditions were 14.5 h light/9.5 h dark. Abbreviations of rice accessions are defined in Table 1. [file 12870_2015_501_MOESM1_ESM.pdf]

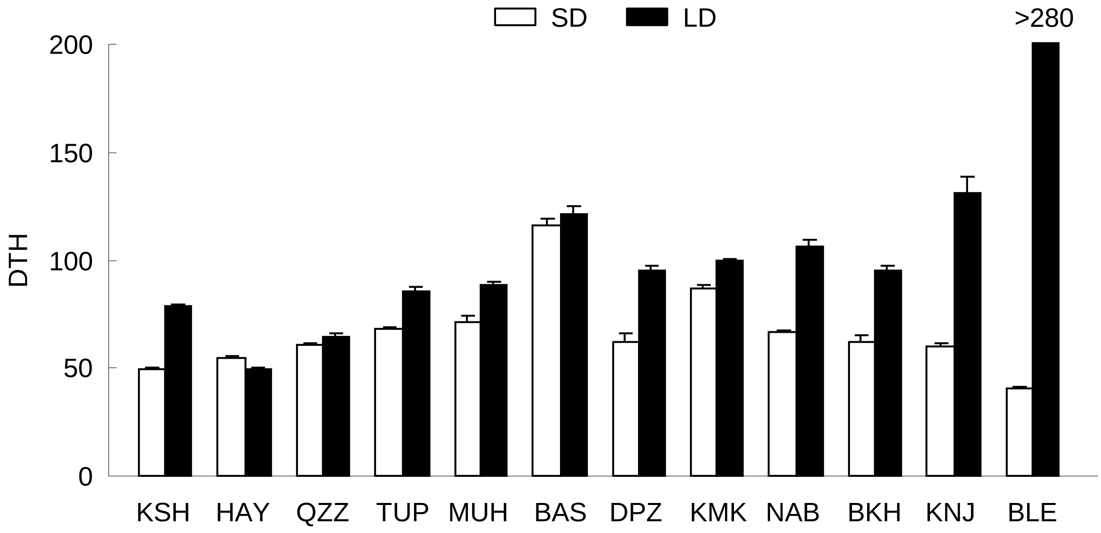

Figure S1. Days to heading (DTH) of Koshihikari (KSH) and 11 diverse accessions of Asian rice under short-day length (SD) and long-day length (LD) conditions. Values are means ± standard deviation (*n* = 10). SD conditions were 10 h light/14 h dark; LD conditions were 14.5 h light/9.5 h dark. Abbreviations of rice accessions are defined in Table 1.
