# Supplementary material for: Genetic architecture of variation in heading date among Asian rice accessions
Source: BMC Plant Biol. 2015 May 8;15:115. doi: 10.1186/s12870-015-0501-x (PMC4424449; doi:10.1186/s12870-015-0501-x)
Supplement: Additional file 2: Figure S2. — Schematic flow chart of development of advanced-backcross populations at BC4F2 and BC4F3 generations. [file 12870_2015_501_MOESM2_ESM.pdf]

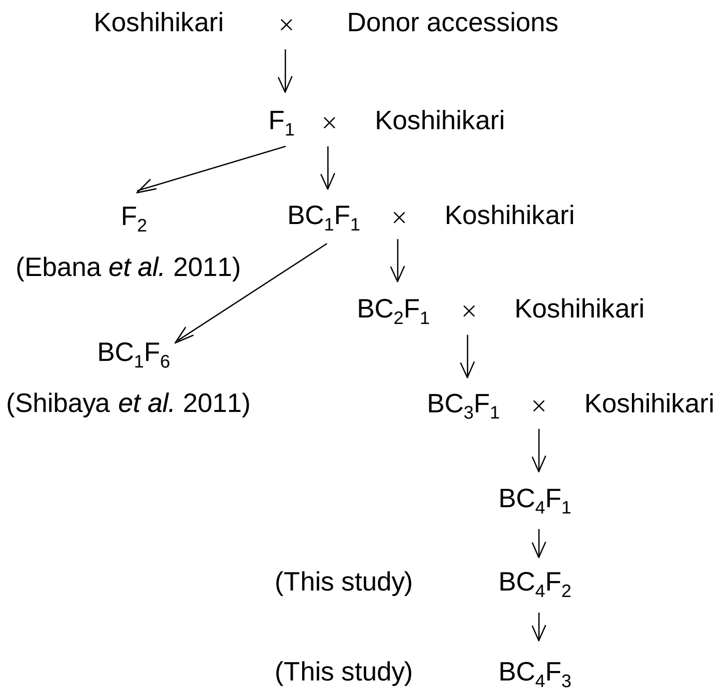

Figure S2. Schematic flow chart of development of advanced-backcross populations at BC_4F_2 and BC_4F_3 generations.
