# Supplementary material for: Genetic architecture of variation in heading date among Asian rice accessions
Source: BMC Plant Biol. 2015 May 8;15:115. doi: 10.1186/s12870-015-0501-x (PMC4424449; doi:10.1186/s12870-015-0501-x)
Supplement: Additional file 3: Figure S3. — Graphical representations of genotypes of 366 BC4F2 populations derived from crosses between Koshihikari (KSH) and 11 diverse accessions of Asian rice. Abbreviations of rice accessions are defined in Table 1. Each horizontal bar corresponds to a rice chromosome; chromosomes are arranged from 1 (left) to 12 (right) with each cell indicating one chromosome of a single population. Each horizontal row indicates the genotype of a BC4F2 population. Regions heterozygous are shown in black, those homozygous for KSH alleles are shown in white, and those missing alleles are shown in gray. Graphical genotypes are based on physical distances according to IRGSP 1.0 of the rice genome sequence [52,53]. [file 12870_2015_501_MOESM3_ESM.pdf]

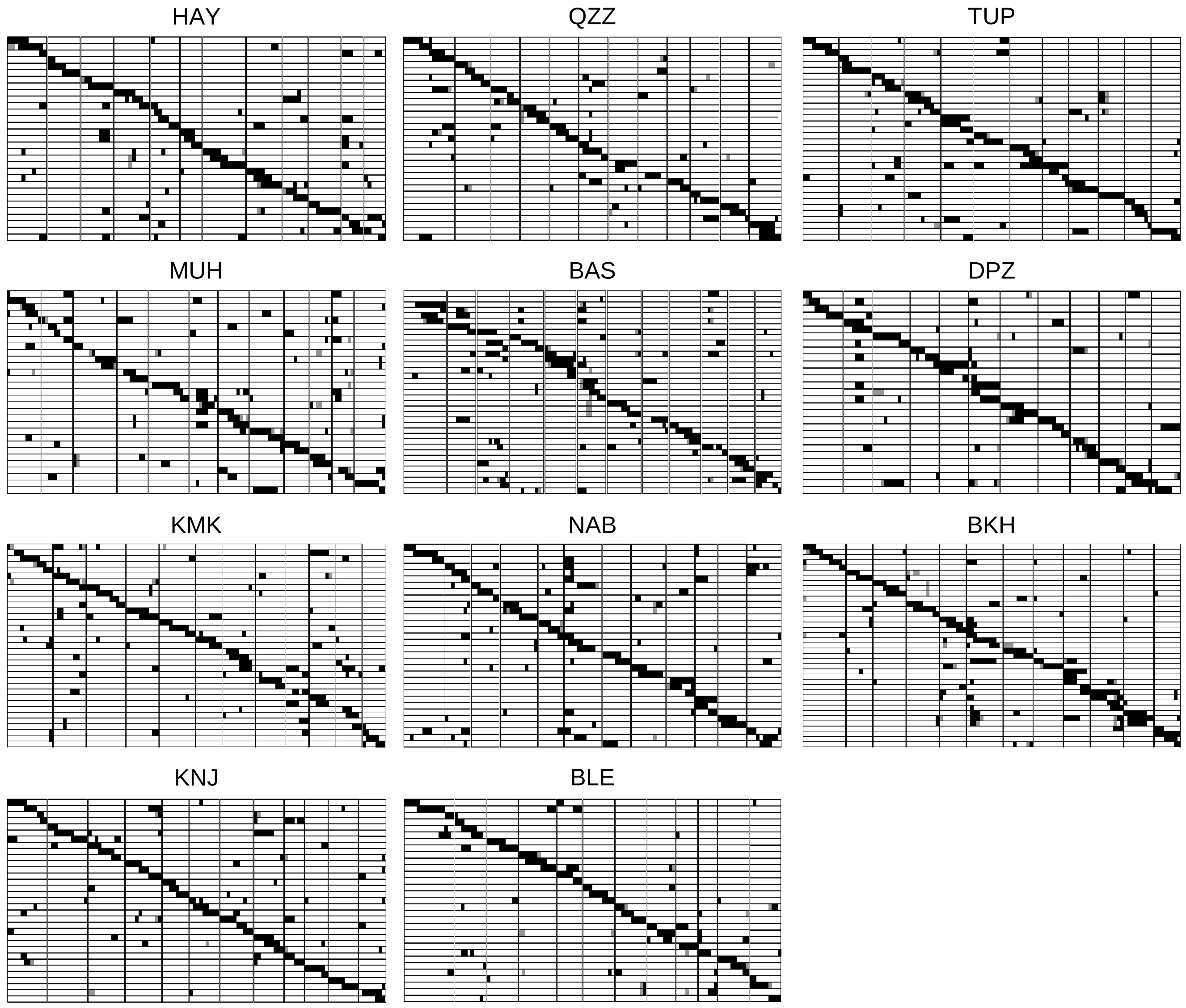

Figure S3. Graphical representations of genotypes of 366 BC_4F_2 populations derived from crosses between Koshihikari (KSH) and 11 diverse accessions of Asian rice. Abbreviations of rice accessions are defined in Table 1. Each horizontal bar corresponds to a rice chromosome; chromosomes are arranged from 1 (left) to 12 (right) with each cell indicating one chromosome of a single population. Each horizontal row indicates the genotype of a BC_4F_2 population. Regions heterozygous are shown in black, those homozygous for KSH alleles are shown in white, and those missing alleles are shown in gray. Graphical genotypes are based on physical distances according to IRGSP 1.0 of the rice genome sequence [52,53].
