# Supplementary material for: Genetic architecture of variation in heading date among Asian rice accessions
Source: BMC Plant Biol. 2015 May 8;15:115. doi: 10.1186/s12870-015-0501-x (PMC4424449; doi:10.1186/s12870-015-0501-x)
Supplement: Additional file 4: Table S1. — Frequency distributions for days to heading (DTH) in 366 BC4F2 populations derived from crosses between Koshihikari (KSH) and 11 diverse accessions of Asian rice. Fill in cells show the number of individual plants: less than five (right pink) and more than six (dark pink). Abbreviations of rice accessions are defined in Table 1. [file 12870_2015_501_MOESM4_ESM.pdf]

Table S1. Frequency distributions for days to heading (DTH) in 366 BC$_4$F$_2$ populations derived from crosses between Koshihikari (KSH) and 11 diverse accessions of Asian rice. Fill in cells show the number of individual plants: less than five (right pink) and more than six (dark pink). Abbreviations of rice accessions are defined in Table 1.

HAY

DTH	KSH	HAY	BC$_4$F$_2$ population																														
			10-2222	10-2223	10-2224	10-2225	10-2226	10-2227	10-2228	10-2229	10-2230	10-2231	10-2232	10-2233	10-2234	10-2236	10-2237	10-2238	10-2239	10-2240	10-2241	10-2242	10-2243	10-2244	10-2245	10-2246	10-2247	10-2248	10-2249	10-2250	10-2251	10-2252	10-2253
85≤	0	24	0	0	0	0	0	0	0	0	0	0	0	0	0	0	0	0	0	0	0	0	0	0	0	0	0	0	0	0	0	0	0
88	0	0	0	0	0	0	0	0	0	0	0	0	0	0	0	1	0	0	0	3	10	0	0	0	0	0	0	0	0	0	0	0	0
91	0	0	0	0	0	0	0	0	0	0	0	0	0	0	0	5	0	0	0	0	1	0	0	2	0	0	0	0	0	0	0	0	0
94	0	0	0	0	0	0	0	0	0	0	0	0	0	0	0	0	0	0	0	0	0	0	4	5	0	0	0	0	0	0	0	0	0
97	0	0	0	0	0	0	0	0	1	0	0	0	0	0	0	0	0	3	0	1	2	0	2	0	0	0	0	0	0	0	0	0	2
100	6	0	4	2	5	9	3	1	16	2	2	7	1	2	0	12	5	3	7	16	7	13	0	2	3	2	5	14	1	6	9	13	5
103	18	0	19	22	17	15	19	11	7	5	20	17	4	18	22	5	1	3	16	4	3	6	14	15	21	16	19	10	3	18	14	11	9
106	0	0	1	0	2	0	2	11	0	0	2	0	0	4	2	1	2	2	1	0	1	5	4	0	0	5	0	0	0	0	1	0	1
109	0	0	0	0	0	0	0	0	0	8	0	0	5	0	0	0	9	3	0	0	0	0	0	0	0	1	0	0	0	0	0	0	3
112	0	0	0	0	0	0	0	0	0	6	0	0	8	0	0	0	6	6	0	0	0	0	0	0	0	0	0	0	15	0	0	0	2
115	0	0	0	0	0	0	0	0	0	3	0	0	4	0	0	0	1	2	0	0	0	0	0	0	0	0	0	0	2	0	0	0	0
118	0	0	0	0	0	0	0	0	0	0	0	0	2	0	0	0	0	2	0	0	0	0	0	0	0	0	0	0	3	0	0	0	2
121	0	0	0	0	0	0	0	0	0	0	0	0	0	0	0	0	0	0	0	0	0	0	0	0	0	0	0	0	0	0	0	0	0
124	0	0	0	0	0	0	0	0	0	0	0	0	0	0	0	0	0	0	0	0	0	0	0	0	0	0	0	0	0	0	0	0	0
127	0	0	0	0	0	0	0	0	0	0	0	0	0	0	0	0	0	0	0	0	0	0	0	0	0	0	0	0	0	0	0	0	0
130	0	0	0	0	0	0	0	0	0	0	0	0	0	0	0	0	0	0	0	0	0	0	0	0	0	0	0	0	0	0	0	0	0
133	0	0	0	0	0	0	0	0	0	0	0	0	0	0	0	0	0	0	0	0	0	0	0	0	0	0	0	0	0	0	0	0	0
136	0	0	0	0	0	0	0	0	0	0	0	0	0	0	0	0	0	0	0	0	0	0	0	0	0	0	0	0	0	0	0	0	0
139	0	0	0	0	0	0	0	0	0	0	0	0	0	0	0	0	0	0	0	0	0	0	0	0	0	0	0	0	0	0	0	0	0
142	0	0	0	0	0	0	0	0	0	0	0	0	0	0	0	0	0	0	0	0	0	0	0	0	0	0	0	0	0	0	0	0	0
145	0	0	0	0	0	0	0	0	0	0	0	0	0	0	0	0	0	0	0	0	0	0	0	0	0	0	0	0	0	0	0	0	0
148	0	0	0	0	0	0	0	0	0	0	0	0	0	0	0	0	0	0	0	0	0	0	0	0	0	0	0	0	0	0	0	0	0

QZZ

DTH	KSH	QZZ	BC$_4$F$_2$ population																																
			11-2108	11-2109	11-2110	11-2111	11-2112	11-2113	11-2114	11-2115	11-2116	11-2117	11-2118	11-2119	11-2120	11-2121	11-2122	11-2123	11-2125	11-2126	11-2127	11-2128	11-2129	11-2130	11-2131	11-2132	11-2133	11-2134	11-2135	11-2136	11-2137	11-2138	11-2139	11-2140	11-2141
85≤	0	0	0	0	0	0	0	0	0	0	0	0	0	0	0	0	0	0	0	0	0	0	0	0	0	0	0	0	0	0	0	0	0	0	0
88	0	4	0	0	0	0	0	0	0	0	0	0	0	0	0	0	0	0	0	0	0	0	0	0	0	0	0	0	0	0	0	0	0	0	0
91	0	2	0	0	0	0	0	0	0	0	1	0	0	0	7	0	0	8	4	0	2	0	3	8	0	6	0	0	0	0	0	0	0	0	0
94	0	0	0	0	0	0	0	0	0	0	2	2	0	0	5	0	0	4	3	0	6	0	0	0	2	5	0	0	0	0	0	0	0	0	0
97	0	0	0	0	0	0	0	0	0	0	2	5	0	0	1	0	0	2	4	0	1	0	0	0	2	3	0	0	0	0	0	1	0	0	0
100	0	0	1	0	0	0	0	0	3	1	6	1	0	1	1	1	0	4	4	0	5	1	0	2	1	5	0	0	0	0	0	0	0	0	0
103	6	0	9	6	7	3	3	7	14	3	5	1	0	4	6	13	0	1	2	0	0	8	1	6	4	2	1	7	5	2	2	2	1	1	0
106	13	0	4	10	9	10	10	13	5	10	3	3	0	15	0	5	0	2	3	10	5	11	3	8	9	3	4	10	9	7	10	9	1	7	6
109	3	0	5	6	6	5	9	2	0	5	1	4	1	2	0	0	3	0	3	9	0	4	7	0	6	0	13	5	0	15	11	8	17	11	13
112	0	0	3	0	0	1	0	0	0	3	1	4	2	0	0	0	16	0	1	0	1	0	9	0	0	0	6	0	1	0	1	1	3	4	3
115	0	0	0	0	0	0	0	0	0	0	1	0	1	0	0	0	2	0	0	0	0	0	1	0	0	0	0	0	0	0	0	0	0	1	0
118	0	0	0	0	0	0	0	0	0	0	0	0	0	0	0	0	0	0	0	0	0	0	0	0	0	0	0	0	0	0	0	0	0	0	0
121	0	0	0	0	0	0	0	0	0	0	0	0	5	0	0	0	0	0	0	0	0	0	0	0	0	0	0	0	0	0	0	0	0	0	0
124	0	0	0	0	0	0	0	0	0	0	0	0	4	0	0	0	0	0	0	0	0	0	0	0	0	0	0	0	0	0	0	0	0	0	0
127	0	0	0	0	0	0	0	0	0	0	0	0	5	0	0	0	0	0	0	0	0	0	0	0	0	0	0	0	0	0	0	0	0	0	0
130	0	0	0	0	0	0	0	0	0	0	0	0	4	0	0	0	0	0	0	0	0	0	0	0	0	0	0	0	0	0	0	0	0	0	0
133	0	0	0	0	0	0	0	0	0	0	0	0	0	0	0	0	0	0	0	0	0	0	0	0	0	0	0	0	0	0	0	0	0	0	0
136	0	0	0	0	0	0	0	0	0	0	0	0	0	0	0	0	0	0	0	0	0	0	0	0	0	0	0	0	0	0	0	0	0	0	0
139	0	0	0	0	0	0	0	0	0	0	0	0	0	0	0	0	0	0	0	0	0	0	0	0	0	0	0	0	0	0	0	0	0	0	0
142	0	0	0	0	0	0	0	0	0	0	0	0	0	0	0	0	0	0	0	0	0	0	0	0	0	0	0	0	0	0	0	0	0	0	0
145	0	0	0	0	0	0	0	0	0	0	0	0	0	0	0	0	0	0	0	0	0	0	0	0	0	0	0	0	0	0	0	0	0	0	0
148	0	0	0	0	0	0	0	0	0	0	0	0	0	0	0	0	0	0	0	0	0	0	0	0	0	0	0	0	0	0	0	0	0	0	0

TUP

DTH	KSH	TUP	BC$_4$F$_2$ population																																	
			10-2151	10-2152	10-2153	10-2154	10-2155	10-2156	10-2157	10-2158	10-2159	10-2160	10-2161	10-2162	10-2163	10-2164	10-2165	10-2166	10-2167	10-2168	10-2169	10-2170	10-2171	10-2172	10-2173	10-2174	10-2175	10-2176	10-2177	10-2178	10-2179	10-2180	10-2181	10-2182	10-2183	10-2184
85≤	0	0	0	0	0	0	0	0	0	0	0	0	0	0	0	0	0	0	0	0	0	0	0	0	0	0	0	0	0	0	0	0	0	0	0	0
88	0	0	0	0	0	0	0	0	0	0	0	0	0	0	0	0	0	0	0	0	0	0	0	0	0	0	0	0	0	0	0	0	0	0	0	0
91	0	0	0	0	0	0	0	0	0	0	0	0	0	0	0	0	0	0	0	0	0	0	0	0	0	0	0	0	0	0	0	0	0	0	0	0
94	0	0	0	0	0	0	0	0	0	0	0	0	0	0	0	0	0	0	0	6	0	0	0	0	0	0	0	0	0	0	0	0	0	0	0	0
97	0	10	0	0	0	0	0	0	0	0	0	0	0	0	0	0	0	0	0	3	0	0	0	0	0	0	0	0	0	0	0	0	0	0	0	0
100	9	11	1	3	2	7	3	0	0	0	0	5	0	2	3	0	2	0	0	12	0	0	0	0	2	0	6	4	1	0	9	3	1	3	0	0
103	13	1	2	18	11	14	17	8	3	4	0	17	0	12	17	10	18	5	17	1	9	5	3	0	13	16	16	18	13	2	12	19	18	16	10	5
106	0	0	1	1	7	1	2	9	5	5	0	0	6	8	2	12	2	9	5	0	11	16	0	2	7	6	0	0	7	18	1	0	3	3	11	13
109	0	0	0	0	2	0	0	5	7	7	0	0	12	0	0	0	0	5	0	0	2	1	1	0	0	0	0	0	1	2	0	0	0	0	1	3
112	0	0	1	0	0	0	0	0	4	4	0	0	2	0	0	0	0	3	0	0	0	0	1	1	0	0	0	0	0	0	0	0	0	0	0	0
115	0	0	1	0	0	0	0	0	3	2	0	0	2	0	0	0	0	0	0	0	0	0	0	0	0	0	0	0	0	0	0	0	0	0	0	0
118	0	0	8	0	0	0	0	0	0	0	8	0	0	0	0	0	0	0	0	0	0	0	0	3	0	0	0	0	0	0	0	0	0	0	0	0
121	0	0	6	0	0	0	0	0	0	0	7	0	0	0	0	0	0	0	0	0	0	0	3	1	0	0	0	0	0	0	0	0	0	0	0	0
124	0	0	1	0	0	0	0	0	0	0	4	0	0	0	0	0	0	0	0	0	0	0	5	1	0	0	0	0	0	0	0	0	0	0	0	0
127	0	0	0	0	0	0	0	0	0	0	2	0	0	0	0	0	0	0	0	0	0	0	4	1	0	0	0	0	0	0	0	0	0	0	0	0
130	0	0	0	0	0	0	0	0	0	0	1	0	0	0	0	0	0	0	0	0	0	0	2	1	0	0	0	0	0	0	0	0	0	0	0	0
133	0	0	0	0	0	0	0	0	0	0	0	0	0	0	0	0	0	0	0	0	0	0	1	4	0	0	0	0	0	0	0	0	0	0	0	0
136	0	0	0	0	0	0	0	0	0	0	0	0	0	0	0	0	0	0	0	0	0	0	2	2	0	0	0	0	0	0	0	0	0	0	0	0
139	0	0	0	0	0	0	0	0	0	0	0	0	0	0	0	0	0	0	0	0	0	0	0	0	0	0	0	0	0	0	0	0	0	0	0	0
142	0	0	0	0	0	0	0	0	0	0	0	0	0	0	0	0	0	0	0	0	0	0	0	3	0	0	0	0	0	0	0	0	0	0	0	0
145	0	0	0	0	0	0	0	0	0	0	0	0	0	0	0	0	0	0	0	0	0	0	0	0	0	0	0	0	0	0	0	0	0	0	0	0
148	0	0	0	0	0	0	0	0	0	0	0	0	0	0	0	0	0	0	0	0	0	0	0	3	0	0	0	0	0	0	0	0	0	0	0	0

MUH

DTH	KSH	MUH	BC$_4$F$_2$ population																														
			10-2341	10-2342	10-2343	10-2344	10-2345	10-2346	10-2347	10-2348	10-2349	10-2350	10-2351	10-2352	10-2353	10-2354	10-2355	10-2356	10-2357	10-2358	10-2359	10-2360	10-2361	10-2362	10-2363	10-2364	10-2365	10-2366	10-2369	10-2370	10-2371	10-2372	10-2373
85≤	0	0	0	0	0	0	0	0	0	0	0	0	0	0	0	0	0	0	0	0	0	0	0	0	0	0	0	0	0	0	0	0	0
88	0	0	0	0	0	0	0	0	0	0	0	0	0	0	0	0	0	0	0	0	0	0	0	0	0	0	0	0	0	0	0	0	0
91	0	0	0	2	0	0	0	0	0	0	0	0	0	0	0	0	0	0	0	4	1	0	1	0	0	0	0	0	0	0	0	8	0
94	0	0	0	3	0	0	0	0	0	0	0	0	0	0	0	0	0	7	4	4	3	0	5	0	0	0	0	0	0	0	0	2	0
97	0	0	0	13	0	0	0	0	0	0	0	0	0	0	0	0	0	5	3	9	12	0	1	0	0	0	0	0	0	0	0	6	0
100	5	1	0	1	3	0	0	2	0	0	0	0	0	0	4	0	3	6	7	2	1	0	8	0	7	8	11	1	0	1	3	0	0
103	18	10	17	5	21	3	20	15	13	8	5	11	2	2	20	17	17	1	2	5	6	11	4	1	17	16	13	5	3	6	14	3	3
106	0	13	6	0	0	2	4	6	11	14	11	10	4	1	0	7	4	0	5	0	1	13	0	8	0	0	0	12	10	13	2	1	2
109	0	0	0	0	0	11	0	0	0	2	6	3	1	1	0	0	0	4	3	0	0	0	2	0	0	0	0	6	7	0	1	0	9
112	0	0	0	0	0	5	0	1	0	0	2	0	0	0	0	0	0	1	0	0	0	0	2	11	0	0	0	0	0	0	0	0	5
115	0	0	0	0	0	3	0	0	0	0	0	0	0	0	0	0	0	0	0	0	0	0	0	2	0	0	0	0	0	0	0	0	1
118	0	0	0	0	0	0	0	0	0	0	0	0	2	2	0	0	0	0	0	0	0	0	1	2	0	0	0	0	0	0	0	0	0
121	0	0	0	0	0	0	0	0	0	0	0	0	11	4	0	0	0	0	0	0	0	0	0	0	0	0	0	0	0	0	0	0	0
124	0	0	0	0	0	0	0	0	0	0	0	0	4	10	0	0	0	0	0	0	0	0	0	0	0	0	0	0	0	0	0	0	0
127	0	0	0	0	0	0	0	0	0	0	0	0	0	2	0	0	0	0	0	0	0	0	0	0	0	0	0	0	0	0	0	0	0
130	0	0	0	0	0	0	0	0	0	0	0	0	0	2	0	0	0	0	0	0	0	0	0	0	0	0	0	0	0	0	0	0	0
133	0	0	0	0	0	0	0	0	0	0	0	0	0	0	0	0	0	0	0	0	0	0	0	0	0	0	0	0	0	0	0	0	0
136	0	0	0	0	0	0	0	0	0	0	0	0	0	0	0	0	0	0	0	0	0	0	0	0	0	0	0	0	0	0	0	0	0
139	0	0	0	0	0	0	0	0	0	0	0	0	0	0	0	0	0	0	0	0	0	0	0	0	0	0	0	0	0	0	0	0	0
142	0	0	0	0	0	0	0	0	0	0	0	0	0	0	0	0	0	0	0	0	0	0	0	0	0	0	0	0	0	0	0	0	0
145	0	0	0	0	0	0	0	0	0	0	0	0	0	0	0	0	0	0	0	0	0	0	0	0	0	0	0	0	0	0	0	0	0
148	0	0	0	0	0	0	0	0	0	0	0	0	0	0	0	0	0	0	0	0	0	0	0	0	0	0	0	0	0	0	0	0	0

BAS

DTH	KSH	BAS	BC$_4$F$_2$ population																																				
			10-2302	10-2303	10-2304	10-2305	10-2306	10-2307	10-2308	10-2309	10-2310	10-2311	10-2312	10-2313	10-2314	10-2315	10-2316	10-2317	10-2318	10-2319	10-2320	10-2321	10-2322	10-2323	10-2324	10-2325	10-2326	10-2327	10-2328	10-2329	10-2330	10-2331	10-2332	10-2333	10-2334	10-2335	10-2336	10-2337	10-2338
85≤	0	0	0	0	0	0	0	0	0	0	0	0	0	0	0	0	0	0	0	0	0	0	0	0	0	0	0	0	0	0	0	0	0	0	0	0	0	0	0
88	0	0	0	0	0	0	0	0	0	0	0	0	0	0	0	0	0	0	0	0	0	0	0	0	0	0	0	0	0	0	0	0	0	0	0	0	0	0	0
91	0	0	0	0	0	0	0	0	0	0	0	0	0	0	0	0	1	0	0	4	0	0	0	0	2	0	0	0	0	0	0	0	0	0	0	0	0	0	0
94	0	0	0	0	0	0	0	0	0	0	0	0	0	0	0	0	2	0	4	4	0	0	0	0	7	0	0	0	0	0	0	0	0	3	0	0	0	0	0
97	0	0	0	0	0	0	0	0	0	0	0	0	0	0	0	0	4	0	11	9	0	0	0	0	4	0	0	0	0	0	0	0	0	4	0	0	0	0	0
100	0	0	1	2	0	2	0	2	3	0	7	0	3	0	0	1	9	0	2	1	1	4	0	0	4	0	2	4	0	0	0	4	0	5	4	0	0	0	1
103	20	0	20	20	17	16	12	16	21	0	17	5	4	1	5	17	0	1	3	5	19	10	13	13	3	13	19	18	7	7	5	19	23	5	18	12	2	3	18
106	4	6	3	2	6	6	11	6	0	0	0	14	0	6	2	6	3	12	3	1	4	5	10	10	2	11	3	2	15	14	16	1	1	1	2	12	2	4	5
109	0	17	0	0	1	0	1	0	0	2	0	5	0	6	0	0	3	11	0	0	0	0	1	1	2	0	0	0	0	3	3	0	0	4	0	0	0	0	0
112	0	1	0	0	0	0	0	0	0	5	0	0	0	6	0	0	2	0	1	0	0	0	0	0	0	0	0	0	0	0	0	0	0	2	0	0	0	0	0
115	0	0	0	0	0	0	0	0	0	9	0	0	1	5	1	0	0	0	0	0	0	0	0	0	0	0	0	0	0	0	0	0	0	0	0	0	0	1	0
118	0	0	0	0	0	0	0	0	0	5	0	0	4	0	0	0	0	0	0	0	0	0	0	0	0	0	0	0	0	0	0	0	0	0	0	0	7	1	0
121	0	0	0	0	0	0	0	0	0	3	0	0	9	0	7	0	0	0	0	0	0	0	0	0	0	0	0	0	0	0	0	0	0	0	0	0	9	8	0
124	0	0	0	0	0	0	0	0	0	0	0	0	1	0	5	0	0	0	0	0	0	0	0	0	0	0	0	0	0	0	0	0	0	0	0	0	4	6	0
127	0	0	0	0	0	0	0	0	0	0	0	0	2	0	3	0	0	0	0	0	0	0	0	0	0	0	0	0	0	0	0	0	0	0	0	0	0	1	0
130	0	0	0	0	0	0	0	0	0	0	0	0	0	0	0	0	0	0	0	0	0	0	0	0	0	0	0	0	0	0	0	0	0	0	0	0	0	0	0
133	0	0	0	0	0	0	0	0	0	0	0	0	0	0	1	0	0	0	0	0	0	0	0	0	0	0	0	0	0	0	0	0	0	0	0	0	0	0	0
136	0	0	0	0	0	0	0	0	0	0	0	0	0	0	0	0	0	0	0	0	0	0	0	0	0	0	0	0	0	0	0	0	0	0	0	0	0	0	0
139	0	0	0	0	0	0	0	0	0	0	0	0	0	0	0	0	0	0	0	0	0	0	0	0	0	0	0	0	0	0	0	0	0	0	0	0	0	0	0
142	0	0	0	0	0	0	0	0	0	0	0	0	0	0	0	0	0	0	0	0	0	0	0	0	0	0	0	0	0	0	0	0	0	0	0	0	0	0	0
145	0	0	0	0	0	0	0	0	0	0	0	0	0	0	0	0	0	0	0	0	0	0	0	0	0	0	0	0	0	0	0	0	0	0	0	0	0	0	0
148	0	0	0	0	0	0	0	0	0	0	0	0	0	0	0	0	0	0	0	0	0	0	0	0	0	0	0	0	0	0	0	0	0	0	0	0	0	0	0

DPZ

DTH	KSH	DPZ	BC$_4$F$_2$ population																												
			11-2004	11-2005	11-2006	11-2007	11-2008	11-2009	11-2010	11-2011	11-2012	11-2013	11-2014	11-2015	11-2016	11-2017	11-2018	11-2019	11-2020	11-2021	11-2022	11-2023	11-2024	11-2025	11-2026	11-2027	11-2028	11-2029	11-2030	11-2031	11-2032
85≤	0	0	0	0	0	0	0	0	0	0	0	0	0	0	0	0	0	0	0	0	0	0	0	0	0	0	0	0	0	0	0
88	0	0	0	0	0	0	0	0	0	0	0	0	0	0	0	0	0	0	0	0	0	0	0	0	0	0	0	0	0	0	0
91	0	0	0	0	0	0	0	0	0	0	0	0	0	0	0	0	0	0	0	0	0	0	0	0	0	0	0	0	0	0	0
94	0	0	0	0	0	0	0	0	0	0	0	0	0	0	0	0	0	0	0	0	0	0	0	0	0	0	0	0	0	0	0
97	0	0	0	0	0	0	0	0	0	0	0	0	0	0	0	0	0	0	0	0	1	0	0	0	0	0	0	0	0	0	0
100	0	0	4	0	0	0	0	0	0	0	0	1	0	0	0	0	0	0	0	0	3	0	1	0	0	1	0	5	0	0	1
103	7	0	12	2	2	0	1	3	0	2	6	6	0	0	0	1	1	5	0	0	3	0	9	4	0	7	1	7	0	0	4
106	12	0	8	1	12	3	4	8	1	2	10	12	0	11	1	2	3	10	7	1	0	2	13	14	1	13	13	10	3	0	12
109	5	0	0	0	10	9	2	8	4	0	4	1	1	8	0	1	1	9	3	0	0	20	1	6	1	3	10	2	1	0	7
112	0	0	0	0	0	10	0	5	10	0	0	2	1	4	1	0	0	0	14	5	10	2	0	0	7	0	0	0	0	7	0
115	0	0	0	4	0	2	2	0	9	0	4	2	1	0	1	2	5	0	0	3	6	0	0	0	2	0	0	0	4	6	0
118	0	2	0	4	0	0	9	0	0	0	0	0	10	0	12	1	11	0	0	6	1	0	0	0	2	0	0	0	11	2	0
121	0	13	0	4	0	0	5	0	0	3	0	0	7	0	8	9	3	0	0	4	0	0	0	0	3	0	0	0	5	7	0
124	0	9	0	3	0	0	0	0	0	8	0	0	4	0	1	1	0	0	0	1	0	0	0	0	2	0	0	0	0	1	0
127	0	0	0	2	0	0	0	0	0	6	0	0	0	0	0	0	0	0	0	4	0	0	0	0	1	0	0	0	0	1	0
130	0	0	0	4	0	0	1	0	0	3	0	0	0	0	0	2	0	0	0	0	0	0	0	0	1	0	0	0	0	0	0
133	0	0	0	0	0	0	0	0	0	0	0	0	0	0	0	2	0	0	0	0	0	0	0	0	0	0	0	0	0	0	0
136	0	0	0	0	0	0	0	0	0	0	0	0	0	0	0	2	0	0	0	0	0	0	0	0	0	0	0	0	0	0	0
139	0	0	0	0	0	0	0	0	0	0	0	0	0	0	0	1	0	0	0	0	0	0	0	0	0	0	0	0	0	0	0
142	0	0	0	0	0	0	0	0	0	0	0	0	0	0	0	0	0	0	0	0	0	0	0	0	0	0	0	0	0	0	0
145	0	0	0	0	0	0	0	0	0	0	0	0	0	0	0	0	0	0	0	0	0	0	0	0	0	0	0	0	0	0	0
148	0	0	0	0	0	0	0	0	0	0	0	0	0	0	0	0	0	0	0	0	0	0	0	0	0	0	0	0	0	0	0

KMK

BC$_4$F$_2$ population

DTH	KSH	KMK	10-2083	10-2084	10-2085	10-2086	10-2087	10-2088	10-2089	10-2090	10-2091	10-2092	10-2093	10-2094	10-2095	10-2096	10-2097	10-2098	10-2099	10-2100	10-2101	10-2102	10-2103	10-2104	10-2105	10-2106	10-2107	10-2108	10-2109	10-2110	10-2111	10-2112	10-2113	10-2114	10-2115	10-2116	10-2117
85≤	0	0	0	0	0	0	0	0	0	0	0	0	0	0	0	0	0	0	0	0	0	0	0	1	0	0	0	0	0	0	0	0	0	0	0	0	0
88	0	0	0	0	0	0	0	0	0	0	0	0	0	0	0	0	0	0	0	0	0	0	0	0	0	0	0	0	0	0	0	0	0	0	0	0	0
91	0	0	0	0	0	0	0	0	0	0	0	0	0	0	0	0	0	3	0	0	0	0	0	0	0	0	0	0	0	0	0	0	0	0	0	0	0
94	0	0	0	0	0	0	0	0	0	0	0	0	0	0	0	0	0	0	4	0	0	0	0	0	0	0	0	0	0	0	0	0	0	0	0	0	0
97	0	0	0	0	0	0	1	0	0	0	0	0	0	0	0	0	0	3	5	0	0	0	0	0	0	0	0	0	0	0	0	0	0	0	0	0	0
100	5	0	4	1	0	1	2	5	7	2	3	1	3	16	3	5	8	3	6	8	4	2	0	3	1	5	16	1	6	5	0	7	3	5	0	1	0
103	16	0	15	20	12	15	19	11	14	12	1	1	2	6	16	17	14	13	3	12	18	16	3	18	5	11	5	18	12	16	4	15	17	17	7	7	9
106	1	0	1	0	10	6	0	6	1	7	15	0	0	0	3	0	0	0	3	2	0	4	6	0	16	5	0	3	4	1	6	0	2	0	10	7	7
109	0	0	2	0	0	0	0	0	0	1	3	0	0	0	0	0	0	0	1	0	0	0	12	0	0	1	0	0	0	0	11	0	0	0	1	3	1
112	0	0	0	0	0	0	0	0	0	0	0	1	6	0	0	0	0	0	0	0	0	0	1	0	0	0	0	0	0	0	0	0	0	0	4	4	5
115	0	0	0	0	0	0	0	0	0	0	0	1	7	0	0	0	0	0	0	0	0	0	0	0	0	0	0	0	0	0	0	0	0	0	0	0	0
118	0	12	0	0	0	0	0	0	0	0	0	9	0	0	0	0	0	0	0	0	0	0	0	0	0	0	0	0	0	0	0	0	0	0	0	0	0
121	0	8	0	0	0	0	0	0	0	0	0	6	4	0	0	0	0	0	0	0	0	0	0	0	0	0	0	0	0	0	0	0	0	0	0	0	0
124	0	0	0	0	0	0	0	0	0	0	0	3	0	0	0	0	0	0	0	0	0	0	0	0	0	0	0	0	0	0	0	0	0	0	0	0	0
127	0	0	0	0	0	0	0	0	0	0	0	0	0	0	0	0	0	0	0	0	0	0	0	0	0	0	0	0	0	0	0	0	0	0	0	0	0
130	0	0	0	0	0	0	0	0	0	0	0	0	0	0	0	0	0	0	0	0	0	0	0	0	0	0	0	0	0	0	0	0	0	0	0	0	0
133	0	0	0	0	0	0	0	0	0	0	0	0	0	0	0	0	0	0	0	0	0	0	0	0	0	0	0	0	0	0	0	0	0	0	0	0	0
136	0	0	0	0	0	0	0	0	0	0	0	0	0	0	0	0	0	0	0	0	0	0	0	0	0	0	0	0	0	0	0	0	0	0	0	0	0
139	0	0	0	0	0	0	0	0	0	0	0	0	0	0	0	0	0	0	0	0	0	0	0	0	0	0	0	0	0	0	0	0	0	0	0	0	0
142	0	0	0	0	0	0	0	0	0	0	0	0	0	0	0	0	0	0	0	0	0	0	0	0	0	0	0	0	0	0	0	0	0	0	0	0	0
145	0	0	0	0	0	0	0	0	0	0	0	0	0	0	0	0	0	0	0	0	0	0	0	0	0	0	0	0	0	0	0	0	0	0	0	0	0
148	0	0	0	0	0	0	0	0	0	0	0	0	0	0	0	0	0	0	0	0	0	0	0	0	0	0	0	0	0	0	1	0	0	0	0	0	0

NAB

BC$_4$F$_2$ population

DTH	KSH	NAB	11-2037	11-2038	11-2039	11-2040	11-2041	11-2042	11-2043	11-2044	11-2045	11-2046	11-2047	11-2048	11-2049	11-2050	11-2051	11-2052	11-2053	11-2054	11-2055	11-2056	11-2057	11-2058	11-2059	11-2060	11-2061	11-2062	11-2063	11-2064	11-2065	11-2066	11-2067	11-2068
85≤	0	0	0	0	0	0	0	0	0	0	0	0	0	0	0	0	0	0	0	0	0	0	0	0	0	0	0	0	0	0	0	0	0	0
88	0	0	0	0	0	0	0	0	0	0	0	0	0	0	0	0	0	0	0	0	0	0	0	0	0	0	0	0	0	0	0	0	0	0
91	0	0	0	0	0	0	0	0	0	0	0	0	0	0	0	0	0	3	0	0	0	0	0	0	0	0	0	0	0	0	0	0	0	0
94	0	0	0	0	0	0	0	0	7	0	0	0	0	0	0	0	0	7	4	0	0	0	0	0	0	0	0	0	0	0	0	0	5	0
97	0	0	0	0	0	0	0	0	3	0	0	0	0	0	0	0	0	2	4	0	0	0	0	0	0	0	0	0	0	0	0	0	5	0
100	0	0	0	0	0	0	2	0	1	0	0	0	0	1	0	0	0	1	7	0	0	0	0	0	0	0	0	0	0	0	0	0	4	0
103	8	0	5	4	1	0	9	1	5	2	0	3	5	9	4	1	1	5	3	1	0	0	1	1	2	4	2	1	2	6	3	1	3	3
106	12	0	11	7	7	0	7	8	1	5	1	11	10	11	10	7	4	1	2	3	0	0	4	12	10	12	11	11	12	11	10	5	1	8
109	4	0	8	13	9	2	3	8	1	7	0	8	3	1	6	13	11	0	2	1	0	4	4	9	10	6	7	9	8	5	7	11	2	9
112	0	0	0	0	6	0	0	4	3	8	1	0	4	0	2	1	6	3	0	6	1	4	10	0	0	0	2	1	0	0	2	5	2	2
115	0	0	0	0	0	0	0	1	1	0	1	0	0	0	0	0	0	0	0	10	4	2	3	0	0	0	0	0	0	0	0	0	0	0
118	0	0	0	0	0	0	0	0	0	0	0	0	0	0	0	0	0	0	0	1	5	7	0	0	0	0	0	0	0	0	0	0	0	0
121	0	0	0	0	0	11	0	0	0	0	2	0	0	0	0	0	0	0	0	0	2	5	0	0	0	0	0	0	0	0	0	0	0	0
124	0	0	0	0	0	5	0	0	0	0	4	0	0	0	0	0	0	0	0	0	3	0	0	0	0	0	0	0	0	0	0	0	0	0
127	0	7	0	0	0	4	0	0	0	0	4	0	0	0	0	0	0	0	0	0	3	0	0	0	0	0	0	0	0	0	0	0	0	0
130	0	13	0	0	0	0	0	0	0	0	5	0	0	0	0	0	0	0	0	0	1	0	0	0	0	0	0	0	0	0	0	0	0	0
133	0	3	0	0	0	0	0	0	0	0	1	0	0	0	0	0	0	0	0	0	1	0	0	0	0	0	0	0	0	0	0	0	0	0
136	0	0	0	0	0	0	0	0	0	0	2	0	0	0	0	0	0	0	0	0	2	0	0	0	0	0	0	0	0	0	0	0	0	0
139	0	0	0	0	0	0	0	0	0	0	0	0	0	0	0	0	0	0	0	0	0	0	0	0	0	0	0	0	0	0	0	0	0	0
142	0	0	0	0	0	0	0	0	0	0	0	0	0	0	0	0	0	0	0	0	0	0	0	0	0	0	0	0	0	0	0	0	0	0
145	0	0	0	0	0	0	0	0	0	0	0	0	0	0	0	0	0	0	0	0	0	0	0	0	0	0	0	0	0	0	0	0	0	0
148	0	0	0	0	0	0	0	0	0	0	0	0	0	0	0	0	0	0	0	0	0	0	0	0	0	0	0	0	0	0	0	0	0	0

BKH

BC$_4$F$_2$ population

DTH	KSH	BKH	10-2042	10-2043	10-2044	10-2045	10-2046	10-2047	10-2048	10-2049	10-2050	10-2051	10-2052	10-2053	10-2054	10-2055	10-2056	10-2057	10-2058	10-2059	10-2060	10-2061	10-2062	10-2063	10-2064	10-2065	10-2066	10-2067	10-2068	10-2069	10-2070	10-2071	10-2072	10-2073	10-2074	10-2075	10-2076	10-2077	10-2078	10-2079	10-2080
85≤	0	0	0	0	0	0	0	0	0	0	0	0	0	0	0	0	0	0	0	0	0	0	0	0	0	0	0	0	0	0	0	0	0	0	0	0	0	0	0	0	0
88	0	0	0	0	0	0	0	0	0	0	0	0	0	0	0	0	0	0	0	0	0	0	0	0	0	0	0	0	0	0	0	0	0	0	0	0	0	0	0	0	0
91	0	0	0	0	0	0	0	0	0	0	0	0	0	0	0	0	0	0	0	0	0	0	0	0	0	4	0	0	0	0	0	0	0	0	0	0	0	0	0	0	0
94	0	0	0	0	0	0	0	0	0	0	0	0	0	0	0	0	0	0	0	0	0	0	0	0	0	3	0	0	0	0	0	0	0	0	0	0	0	0	0	0	0
97	0	0	0	0	0	0	0	0	0	0	1	0	0	0	0	0	0	0	0	0	0	0	0	0	2	0	0	0	0	0	0	0	0	0	0	0	0	0	0	0	0
100	7	0	11	1	7	2	10	5	5	1	6	0	4	6	10	14	0	0	1	0	0	2	2	1	4	7	3	5	0	1	0	0	3	2	0	0	0	2	0	3	1
103	15	0	11	2	15	2	12	16	15	2	14	1	4	5	9	8	1	1	1	4	0	13	8	2	7	8	18	14	2	16	2	1	17	5	1	0	2	17	6	17	7
106	0	0	0	1	0	0	0	1	2	0	1	0	5	8	3	0	4	1	6	2	6	7	6	8	0	0	1	3	2	5	9	5	2	4	0	2	2	3	13	2	11
109	0	0	0	0	0	0	0	0	0	11	0	0	9	3	0	0	9	1	5	0	1	0	6	4	0	0	0	0	7	0	9	8	0	3	0	4	1	0	0	0	3
112	0	0	0	7	0	2	0	0	0	8	0	0	0	0	0	0	2	2	5	2	2	0	0	4	1	0	0	0	2	0	2	2	0	7	1	0	2	0	0	0	0
115	0	0	0	7	0	6	0	0	0	0	0	2	0	0	0	0	5	0	4	3	3	0	0	2	0	0	0	0	3	0	0	3	0	1	1	0	3	0	0	0	0
118	0	0	0	4	0	3	0	0	0	0	0	15	0	0	0	0	1	2	0	4	4	0	0	1	2	0	0	0	4	0	0	3	0	0	3	6	4	0	0	0	0
121	0	0	0	0	0	2	0	0	0	0	0	4	0	0	0	0	0	3	0	1	5	0	0	0	3	0	0	0	2	0	0	0	0	0	2	2	1	0	0	0	0
124	0	0	0	0	0	0	0	0	0	0	0	0	0	0	0	0	0	0	0	2	1	0	0	0	1	0	0	0	0	0	0	0	0	0	4	3	0	0	0	0	0
127	0	0	0	0	0	0	0	0	0	0	0	0	0	0	0	0	0	4	0	2	0	0	0	0	1	0	0	0	0	0	0	0	0	0	1	3	1	0	0	0	0
130	0	3	0	0	0	1	0	0	0	0	0	0	0	0	0	0	0	3	0	0	0	0	0	0	0	0	0	0	0	0	0	0	0	0	1	0	1	0	0	0	0
133	0	12	0	0	0	2	0	0	0	0	0	0	0	0	0	0	0	0	0	1	0	0	0	0	0	0	0	0	0	0	0	0	0	0	3	0	0	0	0	0	0
136	0	5	0	0	0	2	0	0	0	0	0	0	0	0	0	0	0	3	0	0	0	0	0	0	0	0	0	0	0	0	0	0	0	0	2	2	4	0	0	0	0
139	0	2	0	0	0	0	0	0	0	0	0	0	0	0	0	0	0	0	0	0	0	0	0	0	0	0	0	0	0	0	0	0	0	0	0	0	1	0	0	0	0
142	0	0	0	0	0	0	0	0	0	0	0	0	0	0	0	0	0	0	0	0	0	0	0	0	0	0	0	0	0	0	0	0	0	0	0	0	0	0	0	0	0
145	0	0	0	0	0	0	0	0	0	0	0	0	0	0	0	0	0	0	0	0	0	0	0	0	0	0	0	0	0	0	0	0	0	0	0	0	0	0	0	0	0
148	0	0	0	0	0	0	0	0	0	0	0	0	0	0	0	0	0	2	0	1	0	0	0	0	0	0	0	0	0	0	0	0	0	0	3	0	0	0	0	0	0

KNJ

DTH	KSH	KNJ	BC_4F_2 population																																
			10-2187	10-2188	10-2189	10-2190	10-2191	10-2192	10-2193	10-2194	10-2195	10-2196	10-2197	10-2198	10-2199	10-2200	10-2201	10-2202	10-2203	10-2204	10-2205	10-2206	10-2207	10-2208	10-2209	10-2010	10-2011	10-2012	10-2013	10-2014	10-2015	10-2016	10-2017	10-2018	10-2019
85≤	0	0	0	0	0	0	0	0	0	0	0	0	0	0	0	0	0	0	0	0	0	0	0	0	0	0	0	0	0	0	0	0	0	0	0
88	0	0	0	0	0	0	0	0	0	0	0	0	0	0	0	0	0	0	0	0	0	0	0	0	0	0	0	0	0	0	0	0	0	0	0
91	0	0	0	0	0	0	0	0	0	0	0	0	0	0	0	0	0	0	0	0	0	0	0	0	0	0	0	0	0	0	0	0	0	0	0
94	0	0	0	0	0	0	0	0	0	0	0	0	0	0	0	0	0	0	0	0	0	0	0	0	0	0	0	0	0	0	0	0	0	0	0
97	0	0	0	0	0	0	0	0	0	0	0	0	0	0	0	0	0	0	0	0	0	0	0	0	0	0	0	0	0	0	0	0	0	0	0
100	1	0	1	0	4	5	2	0	0	6	2	0	4	2	2	3	8	4	1	1	2	0	0	1	0	0	1	4	5	0	10	4	3	3	8
103	20	0	12	20	17	15	18	7	2	14	15	5	18	19	17	19	14	15	6	4	18	15	15	3	5	15	9	12	17	11	8	15	17	11	13
106	1	0	9	2	1	2	2	8	3	2	4	0	0	1	3	0	0	2	0	2	2	6	7	6	8	7	11	6	0	11	3	3	2	8	1
109	0	0	0	0	0	0	0	7	0	0	1	0	0	0	0	0	0	1	0	0	0	1	0	10	9	0	1	0	0	0	0	0	0	0	0
112	0	0	0	0	0	0	0	0	0	0	0	0	0	0	0	0	0	0	4	0	0	0	0	2	0	0	0	0	0	0	0	0	0	0	0
115	0	0	0	0	0	0	0	0	2	0	0	0	0	0	0	0	0	0	4	5	0	0	0	0	0	0	0	0	0	0	0	0	0	0	0
118	0	0	0	0	0	0	0	0	2	0	0	3	0	0	0	0	0	0	6	2	0	0	0	0	0	0	0	0	0	0	0	0	0	0	0
121	0	0	0	0	0	0	0	0	5	0	0	8	0	0	0	0	0	0	1	5	0	0	0	0	0	0	0	0	0	0	0	0	0	0	0
124	0	0	0	0	0	0	0	0	3	0	0	6	0	0	0	0	0	0	0	3	0	0	0	0	0	0	0	0	0	0	0	0	0	0	0
127	0	0	0	0	0	0	0	0	2	0	0	0	0	0	0	0	0	0	0	0	0	0	0	0	0	0	0	0	0	0	0	0	0	0	0
130	0	0	0	0	0	0	0	0	1	0	0	0	0	0	0	0	0	0	0	0	0	0	0	0	0	0	0	0	0	0	0	0	0	0	0
133	0	0	0	0	0	0	0	0	1	0	0	0	0	0	0	0	0	0	0	0	0	0	0	0	0	0	0	0	0	0	0	0	0	0	0
136	0	0	0	0	0	0	0	0	0	0	0	0	0	0	0	0	0	0	0	0	0	0	0	0	0	0	0	0	0	0	0	0	0	0	0
139	0	0	0	0	0	0	0	0	0	0	0	0	0	0	0	0	0	0	0	0	0	0	0	0	0	0	0	0	0	0	0	0	0	0	0
142	0	0	0	0	0	0	0	0	0	0	0	0	0	0	0	0	0	0	0	0	0	0	0	0	0	0	0	0	0	0	0	0	0	0	0
145	0	0	0	0	0	0	0	0	0	0	0	0	0	0	0	0	0	0	0	0	0	0	0	0	0	0	0	0	0	0	0	0	0	0	0
148	0	22	0	0	0	0	0	0	0	0	0	0	0	0	0	0	0	0	0	0	0	0	0	0	0	0	0	0	0	0	0	0	0	0	0

BLE

DTH	KSH	BLE	BC_4F_2 population																														
			11-2073	11-2074	11-2075	11-2076	11-2077	11-2078	11-2079	11-2080	11-2081	11-2082	11-2083	11-2084	11-2085	11-2086	11-2087	11-2088	11-2089	11-2090	11-2091	11-2092	11-2093	11-2094	11-2095	11-2096	11-2097	11-2098	11-2099	11-2100	11-2101	11-2102	11-2103
85≤	0	0	0	0	0	0	0	0	0	0	0	0	0	0	0	0	0	0	0	0	0	0	0	0	0	0	0	0	0	0	0	0	0
88	0	0	0	0	0	0	0	0	0	0	0	0	0	0	0	0	0	0	0	0	0	0	0	0	0	0	0	0	0	0	0	0	0
91	0	0	0	0	0	0	0	0	0	0	0	0	0	0	0	0	0	0	0	0	0	0	0	0	0	0	0	0	0	0	0	0	0
94	0	0	0	0	0	0	0	0	0	0	0	0	0	0	0	0	0	0	0	0	0	0	0	0	0	0	0	0	0	0	0	0	0
97	0	0	0	0	0	0	0	0	0	0	0	0	0	0	0	0	0	0	0	0	0	0	0	0	0	0	0	0	0	0	0	0	0
100	0	0	0	0	2	1	0	0	0	0	0	0	0	0	0	0	0	0	0	0	0	0	0	0	0	0	0	0	0	2	0	0	0
103	5	0	0	1	9	5	3	1	0	0	0	0	1	1	4	2	0	0	0	0	0	0	2	2	3	0	7	1	4	3	0	0	0
106	10	0	6	8	11	6	8	5	1	2	7	9	4	7	8	7	2	1	5	5	2	4	12	11	10	1	10	6	9	7	1	2	0
109	7	0	10	12	0	6	8	13	2	2	12	13	10	6	7	9	3	1	6	4	0	3	8	7	8	12	4	13	7	6	5	6	8
112	0	0	6	1	0	0	3	3	14	2	3	0	2	8	3	4	1	4	11	6	9	1	0	2	1	9	1	2	2	3	11	6	13
115	0	0	0	0	0	0	0	0	3	0	0	0	0	0	0	0	0	0	0	5	11	8	0	0	0	0	0	0	0	1	3	5	1
118	0	0	0	0	0	0	0	0	2	0	0	0	0	0	0	0	9	0	0	2	0	3	0	0	0	0	0	0	0	0	1	0	0
121	0	0	0	0	0	0	0	0	0	10	0	0	0	0	0	0	3	6	0	0	0	3	0	0	0	0	0	0	0	0	0	0	0
124	0	0	0	0	0	0	0	0	0	5	0	0	0	0	0	0	3	9	0	0	0	0	0	0	0	0	0	0	0	0	0	0	0
127	0	0	0	0	0	0	0	0	0	1	0	0	0	0	0	0	1	0	0	0	0	0	0	0	0	0	0	0	0	0	0	0	0
130	0	0	0	0	0	0	0	0	0	0	0	0	0	0	0	0	0	1	0	0	0	0	0	0	0	0	0	0	0	0	0	0	0
133	0	0	0	0	0	0	0	0	0	0	0	0	0	0	0	0	0	0	0	0	0	0	0	0	0	0	0	0	0	0	0	0	0
136	0	0	0	0	0	0	0	0	0	0	0	0	0	0	0	0	0	0	0	0	0	0	0	0	0	0	0	0	0	0	0	0	0
139	0	0	0	0	0	0	0	0	0	0	0	0	0	0	0	0	0	0	0	0	0	0	0	0	0	0	0	0	0	0	0	0	0
142	0	0	0	0	0	0	0	0	0	0	0	0	0	0	0	0	0	0	0	0	0	0	0	0	0	0	0	0	0	0	0	0	0
145	0	0	0	0	0	0	0	0	0	0	0	0	0	0	0	0	0	0	0	0	0	0	0	0	0	0	0	0	0	0	0	0	0
148	0	22	0	0	0	0	0	0	0	0	0	0	0	0	0	0	0	0	0	0	0	0	0	0	0	0	0	0	0	0	0	0	0
