# Supplementary material for: Genetic architecture of variation in heading date among Asian rice accessions
Source: BMC Plant Biol. 2015 May 8;15:115. doi: 10.1186/s12870-015-0501-x (PMC4424449; doi:10.1186/s12870-015-0501-x)
Supplement: Additional file 5: Table S2. — List of heading date QTLs detected in BC4F2 populations derived from crosses between Koshihikari (KSH) and 11 diverse accessions of Asian rice. QTLs written by bold characters indicate those confirmed in BC4F3 populations. Abbreviations of rice accessions are defined in Table 1. [file 12870_2015_501_MOESM5_ESM.pdf]

**Table S2.** List of heading date QTLs detected in $BC_4F_2$ populations derived from crosses between Koshihikari (KSH) and 11 diverse accessions of Asian rice. QTLs written by bold characters indicate those confirmed in $BC_4F_3$ populations. Abbreviations of rice accessions are defined in Table 1.

| Population | | Chromosome | Physical position | Marker interval | LOD[a] | Additive effect[b] | Dominance effect[c] | PVE (%)[d] | Corresponding gene[e] | Located near gene[f] |
|---|---|---|---|---|---|---|---|---|---|---|
| HAY | 10_2237 | 3 | 28.8–33.7 | RM6329–RM1038 | 15.3 | -6.7 | 1.9 | 74.2 | *Hd6* | *Hd16* |
| HAY | 10_2238 | 3 | 28.8–33.7 | RM6329–RM1038 | 6.8 | -7.6 | 0.4 | 59.9 | *Hd6* | *Hd16* |
| HAY | 10_2232 | 3 | 33.7 | RM1038 | 12.4 | -6.0 | 2.1 | 86.9 | *Hd6* | *Hd16* |
| HAY | 10_2249 | 3 | 33.7 | RM1038 | 12.5 | -6.2 | 3.6 | 72.3 | *Hd6* | *Hd16* |
| HAY | 10_2253 | 3 | 33.7 | RM1038 | 4.1 | -4.0 | -2.7 | 24.0 | *Hd6* | *Hd16* |
| HAY | 10_2229 | 3 | 33.7–34.0 | RM1038–RM2187 | 12.2 | -6.2 | 2.3 | 56.6 | *Hd6* | *Hd16* |
| HAY | 10_2237 | 6 | 0.1–10.8 | RM19227–RM19835 | 7.2 | -2.2 | -1.6 | 12.8 | *Hd1* | |
| HAY | 10_2238 | 6 | 10.8–19.7 | RM19835–RM20158 | 3.0 | -3.2 | -2.0 | 15.4 | | |
| HAY | 10_2240 | 7 | 5.0–11.0 | RM21137–RM21392 | 16.7 | 7.3 | 4.3 | 92.0 | *Ghd7* | |
| HAY | 10_2241 | 7 | 5.0–17.3 | RM21137–RM21577 | 16.5 | 7.1 | 3.4 | 52.3 | *Ghd7* | |
| HAY | 10_2242 | 7 | 15.9–21.2 | RM21516–RM21770 | 3.0 | -1.4 | 0.7 | 16.1 | | |
| HAY | 10_2242 | 7 | 24.2–29.4 | RM6326–RM22164 | 9.8 | 2.7 | -1.6 | 81.0 | | *OsPRR37* |
| HAY | 10_2253 | 7 | 28.7–29.4 | RM5720–RM22164 | 8.1 | 5.6 | -4.1 | 50.7 | | *OsPRR37* |
| HAY | 10_2243 | 8 | 0.1–8.6 | RM5911–RM22679 | 10.8 | 4.1 | 4.3 | 83.7 | *DTH8* | |
| HAY | 10_2236 | 8 | 4.5–8.6 | RM22496–RM22679 | 14.0 | 6.5 | 4.1 | 89.3 | *DTH8* | |
| HAY | 10_2244 | 8 | 4.5–8.6 | RM22496–RM22679 | 12.4 | 5.3 | 4.2 | 91.8 | *DTH8* | |

| Population | | Chromosome | Physical position | Marker Interval | LOD[a] | Additive effect[b] | Dominance effect[c] | PVE (%)[d] | Corresponding gene[e] | Located near gene[f] |
|---|---|---|---|---|---|---|---|---|---|---|
| QZZ | 11_2108 | **1** | **0.2–4.1** | **RM6887–RM8105** | **5.7** | **2.2** | **3.9** | **66.3** | | |
| QZZ | 11_2109 | 1 | 19.4–21.9 | RM11069–RM1196 | 2.2 | 0.9 | -2.1 | 34.7 | | |
| QZZ | 11_2122 | 1 | 41.2–42.9 | RM5310–RM8137 | 2.4 | 1.3 | 0.1 | 28.4 | | |
| QZZ | 11_2112 | 2 | 6.7–11.3 | RM5897–RM1234 | 2.0 | -1.3 | -0.6 | 32.8 | | |
| QZZ | 11_2122 | **3** | **0.5–9.9** | **RM4108–RM1371** | **3.4** | **-1.7** | **0.9** | **37.0** | | *Ehd4* , *DTH3* |
| QZZ | 11_2118 | 3 | 5.5–9.9 | RM5442–RM1371 | 4.6 | -5.1 | 2.4 | 9.7 | | |
| QZZ | 11_2118 | **3** | **26.7–35.6** | **RM2334–RM3329** | **7.0** | **-5.1** | **2.0** | **19.1** | *Hd6* | *Hd16* |
| QZZ | 11_2119 | 4 | 4.9–13.1 | RM16449–RM5633 | 2.6 | 1.2 | -1.1 | 39.4 | | |
| QZZ | 11_2121 | 4 | 28.8–32.1 | RM3916–RM2121 | 3.8 | 0.1 | -2.0 | 57.4 | | |
| QZZ | 11_2131 | 6 | 0.2–5.2 | RM6467–RM5754 | 2.0 | -1.5 | 4.2 | 20.1 | *RFT1, Hd3a* | *Hd17* |
| QZZ | 11_2116 | 6 | 8.8 | RM5963 | 5.7 | 7.5 | 0.6 | 66.6 | *Hd1* | |
| QZZ | 11_2120 | 6 | 8.8 | RM5963 | 7.2 | 5.3 | -2.5 | 75.4 | *Hd1* | |
| QZZ | 11_2123 | 6 | 8.8 | RM5963 | 9.9 | 6.5 | -0.5 | 85.5 | *Hd1* | |
| QZZ | 11_2125 | 6 | 8.8 | RM5963 | 3.8 | 6.5 | 2.8 | 50.2 | *Hd1* | |
| QZZ | 11_2127 | 6 | 8.8–20.0 | RM5963–RM20176 | 5.7 | 6.7 | -1.9 | 69.3 | *Hd1* | |
| QZZ | 11_2132 | 6 | 8.8–20.0 | RM5963–RM20176 | 15.9 | 6.2 | -1.2 | 77.4 | *Hd1* | |
| QZZ | 11_2130 | **7** | **3.3–13.4** | **RM1353–RM7273** | **16.4** | **8.2** | **6.1** | **72.3** | *Ghd7* | |
| QZZ | 11_2129 | **7** | **3.3–26.8** | **RM1353–RM1364** | **11.7** | **10.9** | **10.5** | **81.0** | | *OsPRR37* |
| QZZ | 11_2117 | 8 | 0.4–5.9 | RM1381–RM6838 | 3.9 | 5.8 | 3.3 | 34.3 | *DTH8* | |
| QZZ | 11_2131 | 8 | 5.9–12.4 | RM6838–RM22839 | 7.1 | 5.0 | 6.4 | 61.0 | *DTH8* | |
| QZZ | 11_2131 | 8 | 18.8–22.5 | RM5767–RM23251 | 3.9 | -0.9 | 6.0 | 34.0 | | |
| QZZ | 11_2133 | 9 | 15.2–20.5 | RM5122–RM3808 | 2.1 | 1.5 | -0.3 | 31.2 | | |
| QZZ | 11_2135 | 10 | 6.1–7.6 | RM7276–RM25151 | 3.4 | -3.8 | -2.7 | 60.1 | | |
| QZZ | 11_2138 | 10 | 13.4–14.5 | RM25302–RM5147 | 2.6 | -3.4 | 1.1 | 45.3 | | |
| QZZ | 11_2132 | 12 | 1.0–2.3 | RM3323–RM3747 | 2.5 | -1.1 | 0.1 | 2.3 | | |
| QZZ | 11_2139 | 12 | 2.3–20.0 | RM3747–RM28305 | 3.4 | -1.6 | 0.6 | 48.8 | | |

| Population | | Chromo some | Physical position | Marker Interval | LOD[a] | Additive effect[b] | Dominance effect[c] | PVE (%)[d] | Corresponding gene[e] | Located near gene[f] |
|---|---|---|---|---|---|---|---|---|---|---|
| TUP | 10_2153 | 1 | 26.8–31.5 | RM3143–RM5914 | 3.0 | -1.2 | -1.1 | 28.1 | | |
| TUP | 10_2179 | **2** | **1.9** | **RM7562** | **4.9** | **2.1** | **-0.8** | **61.1** | | |
| TUP | 10_2156 | **2** | **6.7–11.3** | **RM5897–RM1234** | **3.8** | **-1.6** | **0.7** | **39.1** | | |
| TUP | 10_2156 | **2** | **30.6–35.4** | **RM3316–RM3850** | **4.1** | **-1.6** | **0.7** | **44.1** | | *DTH2* |
| TUP | 10_2166 | **3** | **0.5** | **RM4108** | **4.3** | **-3.9** | **-1.5** | **54.3** | | *Ehd4* , *DTH3* |
| TUP | 10_2158 | **3** | **0.5–14.5** | **RM4108–RM6959** | **6.4** | **-3.7** | **0.6** | **57.6** | | *Ehd4* , *DTH3* |
| TUP | 10_2158 | 3 | 17.4–25.1 | RM1334–RM3513 | 2.9 | -2.9 | -1.3 | 15.9 | | |
| TUP | 10_2159 | 3 | 28.7–32.4 | RM1350–RM6970 | 6.4 | -2.8 | -2.8 | 70.3 | *Hd6* | *Hd16* |
| TUP | 10_2171 | 3 | 28.7–32.4 | RM1350–RM6970 | 11.8 | -11.2 | 7.3 | 63.2 | *Hd6* | *Hd16* |
| TUP | 10_2172 | 3 | 28.7–32.4 | RM1350–RM6970 | 10.9 | -12.7 | 6.5 | 55.2 | *Hd6* | *Hd16* |
| TUP | 10_2151 | 3 | 32.4–35.6 | RM6970–RM3329 | 5.2 | -6.9 | 7.0 | 64.6 | *Hd6* | *Hd16* |
| TUP | 10_2172 | 5 | 2.9–6.1 | RM6517–RM7118 | 2.2 | 2.0 | 6.1 | 4.2 | | |
| TUP | 10_2166 | **5** | **23.9–29.5** | **RM3476–RM3286** | **2.3** | **-1.1** | **2.0** | **19.1** | | |
| TUP | 10_2168 | 6 | 7.6–15.8 | RM19715–RM20023 | 4.3 | 2.2 | 2.2 | 57.7 | *Hd1* | |
| TUP | 10_2153 | **6** | **20.3–24.5** | **RM7193–RM5957** | **4.6** | **-2.0** | **-1.3** | **47.5** | | |
| TUP | 10_2182 | **6** | **24.5** | **RM5957** | **2.1** | **-1.6** | **-0.4** | **33.8** | | |
| TUP | 10_2169 | 7 | 0.3–3.3 | RM20810–RM1353 | 2.2 | -1.6 | -0.2 | 35.3 | | |
| TUP | 10_2171 | 7 | 23.6–29.4 | RM5847–RM22164 | 2.2 | -3.9 | 1.3 | 10.8 | | *OsPRR37* |
| TUP | 10_2172 | 7 | 23.6–29.4 | RM5847–RM22164 | 2.5 | -5.8 | 1.6 | 7.8 | | *OsPRR37* |
| TUP | 10_2172 | 8 | 0.1–2.9 | RM6369–RM22380 | 4.1 | -1.3 | -2.1 | 15.2 | | |
| TUP | 10_2173 | 8 | 5.9–16.0 | RM6838–RM22934 | 2.3 | -1.2 | 2.4 | 36.0 | *DTH8* | |
| TUP | 10_2174 | 8 | 22.9–27.4 | RM7556–RM3480 | 3.8 | 1.1 | 0.0 | 52.7 | | |
| TUP | 10_2175 | 9 | 7.8–9.2 | RM1328–RM23941 | 2.0 | 0.9 | 0.4 | 32.8 | | |
| TUP | 10_2176 | 9 | 7.8–9.2 | RM1328–RM23941 | 2.8 | 0.8 | 0.0 | 42.2 | | |
| TUP | 10_2163 | 10 | 2.0 | RM24944 | 2.6 | -1.2 | 0.6 | 40.5 | | |
| TUP | 10_2177 | **10** | **11.7–17.4** | **RM4455–RM5620** | **4.4** | **-1.4** | **-1.9** | **55.5** | | *Ehd1* |
| TUP | 10_2181 | 11 | 0.3 | RM5716 | 2.1 | 1.5 | -1.1 | 33.2 | | |
| TUP | 10_2180 | 11 | 10.0–14.5 | RM4862–RM5824 | 2.9 | 0.8 | 0.6 | 25.7 | | |
| TUP | 10_2184 | **12** | **20.0–24.4** | **RM28305–RM5479** | **4.9** | **-0.6** | **-3.1** | **62.5** | | |
| TUP | 10_2178 | **12** | **24.4–27.4** | **RM5479–RM2197** | **3.5** | **-1.3** | **0.5** | **49.2** | | |
| TUP | 10_2168 | 12 | 27.4 | RM2197 | 2.1 | -0.6 | 2.0 | 13.9 | | |

| Population | | Chromo some | Physical position | Marker Interval | LOD[a] | Additive effect[b] | Dominance effect[c] | PVE (%)[d] | Corresponding gene[e] | Located near gene[f] |
|---|---|---|---|---|---|---|---|---|---|---|
| MUH | 10_2369 | **3** | **0.5** | **RM4108** | **8.6** | **-1.6** | **0.1** | **51.9** | | *Ehd4* , *DTH3* |
| MUH | 10_2349 | **3** | **0.5–2.7** | **RM4108–RM14391** | **6.6** | **-3.5** | **-0.4** | **68.8** | | *Ehd4* , *DTH3* |
| MUH | 10_2352 | 3 | 26.7–35.0 | RM2334–RM16153 | 10.8 | -9.3 | 7.0 | 61.0 | *Hd6* | *Hd16* |
| MUH | 10_2351 | 3 | 26.7–35.6 | RM2334–RM3329 | 15.5 | -8.4 | 7.5 | 90.9 | *Hd6* | *Hd16* |
| MUH | 10_2355 | 5 | 8.3–15.8 | RM2744–RM18419 | 3.2 | 1.2 | -0.4 | 44.0 | | |
| MUH | 10_2357 | **5** | **23.9–27.9** | **RM3476–RM5784** | **2.8** | **-1.5** | **0.4** | **5.3** | | |
| MUH | 10_2342 | **6** | **5.2–8.1** | **RM5754–RM19725** | **9.0** | **3.8** | **-3.3** | **78.9** | *Hd1* | |
| MUH | 10_2372 | **6** | **8.1** | **RM19725** | **7.0** | **5.9** | **-1.9** | **76.0** | *Hd1* | |
| MUH | 10_2356 | **6** | **8.1–20.3** | **RM19725–RM7193** | **10.1** | **7.1** | **-2.5** | **82.1** | *Hd1* | |
| MUH | 10_2357 | **6** | **8.1–20.3** | **RM19725–RM7193** | **14.3** | **5.9** | **-0.8** | **78.5** | *Hd1* | |
| MUH | 10_2359 | **6** | **8.1–20.3** | **RM19725–RM7193** | **10.4** | **4.6** | **-2.1** | **82.9** | *Hd1* | |
| MUH | 10_2361 | **6** | **8.1–20.3** | **RM19725–RM7193** | **14.0** | **9.2** | **-1.8** | **89.3** | *Hd1* | |
| MUH | 10_2358 | **6** | **15.8–24.5** | **RM20023–RM5957** | **6.2** | **4.8** | **-0.9** | **69.3** | | |
| MUH | 10_2362 | **8** | **0.1–6.8** | **RM6369–RM22617** | **7.8** | **-5.7** | **0.2** | **74.4** | *DTH8* | |
| MUH | 10_2344 | 8 | 6.8–15.7 | RM22617–RM22921 | 8.4 | -3.9 | -0.1 | 76.5 | | |
| MUH | 10_2373 | 8 | 10.3–19.1 | RM3395–RM23055 | 2.3 | -3.1 | -0.3 | 39.4 | | |
| MUH | 10_2364 | 8 | 28.2 | RM4997 | 2.4 | 0.6 | -0.3 | 27.4 | | |
| MUH | 10_2364 | 9 | 7.5–9.2 | RM1328–RM23928 | 2.5 | 1.1 | -0.4 | 28.7 | | |
| MUH | 10_2369 | 10 | 7.6–11.7 | RM25151–RM4455 | 3.0 | -0.9 | 0.2 | 8.0 | | |
| MUH | 10_2369 | 10 | 17.4–23.1 | RM5620–RM6673 | 4.3 | 0.9 | -0.4 | 12.7 | | *Ehd1* |
| MUH | 10_2348 | **11** | **3.8–8.1** | **RM5599–RM3701** | **2.3** | **-1.3** | **0.7** | **34.5** | | |
| MUH | 10_2370 | 12 | 24.4–27.4 | RM5479–RM2197 | 2.5 | -1.1 | 1.0 | 42.0 | | |

| Population | | Chromo some | Physical position | Marker Interval | LOD[a] | Additive effect[b] | Dominance effect[c] | PVE (%)[d] | Corresponding gene[e] | Located near gene[f] |
|---|---|---|---|---|---|---|---|---|---|---|
| BAS | 10_2304 | **1** | **12.1–18.7** | **RM10764–RM11033** | **3.3** | **-2.6** | **26.6** | **46.7** | | |
| BAS | 10_2307 | 1 | 26.8–31.5 | RM3143–RM5914 | 3.8 | -1.8 | 0.9 | 49.9 | | |
| BAS | 10_2316 | 2 | 18.5–24.0 | RM1211–RM3515 | 2.7 | -3.9 | -6.1 | 27.2 | | |
| BAS | 10_2313 | **2** | **33.0–35.4** | **RM7286–RM3850** | **4.2** | **-2.2** | **-1.3** | **15.2** | | *DTH2* |
| BAS | 10_2316 | **3** | **0.5–3.8** | **RM4108–RM14462** | **2.8** | **0.2** | **5.4** | **17.7** | | *Ehd4* , *DTH3* |
| BAS | 10_2311 | **3** | **10.1–25.1** | **RM14778–RM3513** | **3.4** | **-2.0** | **-0.3** | **44.5** | | |
| BAS | 10_2313 | **3** | **10.1–14.5** | **RM14778–RM6959** | **2.3** | **-1.2** | **-0.9** | **7.0** | | |
| BAS | 10_2312 | 3 | 32.4–35.6 | RM6970–RM3329 | 15.1 | -10.3 | 7.6 | 90.6 | *Hd6* | *Hd16* |
| BAS | 10_2314 | 3 | 32.4–35.6 | RM6970–RM3329 | 11.6 | -10.2 | 7.8 | 85.0 | *Hd6* | *Hd16* |
| BAS | 10_2337 | 3 | 32.4–35.6 | RM6970–RM3329 | 13.1 | -9.3 | 6.9 | 88.1 | *Hd6* | *Hd16* |
| BAS | 10_2315 | 5 | 8.3–10.1 | RM2744–RM18222 | 2.6 | 0.8 | -1.5 | 37.9 | | |
| BAS | 10_2315 | 5 | 14.9–23.9 | RM6742–RM3476 | 2.7 | 0.9 | -1.5 | 38.6 | | |
| BAS | 10_2316 | 5 | 23.9–29.4 | RM3476–RM19193 | 3.1 | -4.9 | -1.1 | 33.3 | | |
| BAS | 10_2317 | 5 | 23.9–29.4 | RM3476–RM19193 | 2.1 | -1.1 | 0.2 | 31.9 | | |
| BAS | 10_2318 | 6 | 5.2–8.8 | RM5754–RM5963 | 3.7 | 6.4 | -2.8 | 48.9 | | |
| BAS | 10_2319 | 6 | 5.2–15.8 | RM5754–RM20023 | 13.1 | 6.1 | -0.9 | 89.3 | *Hd1* | |
| BAS | 10_2323 | 7 | 11.0–16.0 | RM21392–RM21521 | 2.6 | -1.5 | -0.2 | 39.9 | | |
| BAS | 10_2313 | **7** | **29.4** | **RM22164** | **7.8** | **-3.2** | **0.3** | **50.3** | | *OsPRR37* |
| BAS | 10_2318 | 8 | 0.4–5.9 | RM1381–RM6838 | 2.5 | -4.1 | 2.5 | 39.6 | *DTH8* | |
| BAS | 10_2326 | 9 | 3.4–10.5 | RM23736–RM24075 | 2.4 | 1.3 | -0.1 | 35.6 | | |
| BAS | 10_2334 | 11 | 14.5–25.1 | RM5824–RM2191 | 3.5 | 1.1 | 0.7 | 49.2 | | |

| Population | | Chromo some | Physical position | Marker Interval | LOD[a] | Additive effect[b] | Dominance effect[c] | PVE (%)[d] | Corresponding gene[e] | Located near gene[f] |
|---|---|---|---|---|---|---|---|---|---|---|
| DPZ | 11_2004 | 1 | 2.2–7.3 | RM5423–RM3598 | 3.0 | 2.0 | 0.1 | 41.5 | | |
| DPZ | 11_2006 | 1 | 21.9–26.8 | RM1196–RM3143 | 4.1 | -1.9 | 0.8 | 59.3 | | |
| DPZ | 11_2019 | **2** | **13.4–18.4** | **RM13101–RM1211** | **4.1** | **1.0** | **-1.9** | **32.7** | | |
| DPZ | 11_2005 | **2** | **18.4–24.0** | **RM1211–M3515** | **2.7** | **0.6** | **-5.5** | **10.8** | | |
| DPZ | 11_2017 | 2 | 24.0 | RM3515 | 2.1 | 2.7 | -4.5 | 7.1 | | |
| DPZ | 11_2009 | **2** | **29.3–33.0** | **RM6933–RM7286** | **2.3** | **-1.9** | **-1.2** | **34.6** | | *DTH2* |
| DPZ | 11_2007 | **2** | **33.0–35.4** | **RM7286–RM3850** | **5.3** | **-3.0** | **0.2** | **63.2** | | *DTH2* |
| DPZ | 11_2010 | 3 | 0.1–1.5 | RM14243–RM3372 | 3.3 | -1.8 | 1.0 | 44.9 | | *Ehd4* , *DTH3* |
| DPZ | 11_2031 | 3 | 19.2–21.4 | RM15289–RM5488 | 2.6 | -2.4 | 0.8 | 17.3 | | |
| DPZ | 11_2019 | 3 | 25.1 | RM3513 | 3.5 | -1.7 | 0.2 | 41.6 | | |
| DPZ | 11_2031 | 3 | 25.1–27.3 | RM3513–RM6736 | 2.1 | -2.7 | -0.8 | 14.4 | | |
| DPZ | 11_2011 | 3 | 27.3–35.6 | RM6736–RM3329 | 13.0 | -11.7 | 7.7 | 89.2 | *Hd6* | *Hd16* |
| DPZ | 11_2014 | 5 | 23.9–27.9 | RM3476–RM5784 | 2.8 | -1.6 | 1.5 | 15.4 | | |
| DPZ | 11_2013 | **6** | **0.2** | **RM6467** | **3.8** | **-3.1** | **-3.4** | **49.7** | | |
| DPZ | 11_2031 | **6** | **0.2–6.0** | **RM6467–RM2615** | **3.8** | **-2.8** | **-2.7** | **40.2** | *RFT1, Hd3a* | *Hd17* |
| DPZ | 11_2005 | 6 | 0.2–8.1 | RM6467–RM19725 | 7.1 | -11.3 | 1.5 | 71.4 | *Hd1* | |
| DPZ | 11_2014 | 6 | 6.0–8.1 | RM2615–RM19725 | 10.5 | -6.2 | 3.4 | 63.4 | *Hd1* | |
| DPZ | 11_2016 | 6 | 6.0–8.1 | RM2615–RM19725 | 9.6 | -5.7 | 2.2 | 81.5 | *Hd1* | |
| DPZ | 11_2017 | 6 | 6.0–8.1 | RM2615–RM19725 | 8.5 | -11.7 | 0.6 | 75.0 | *Hd1* | |
| DPZ | 11_2018 | 6 | 6.0–8.1 | RM2615–RM19725 | 11.6 | -6.8 | 3.7 | 85.5 | *Hd1* | |
| DPZ | 11_2008 | 6 | 8.1 | RM19725 | 10.4 | -8.0 | 3.0 | 82.8 | *Hd1* | |
| DPZ | 11_2026 | 6 | 8.1–11.0 | RM19725–RM3330 | 6.4 | -6.7 | 1.5 | 73.0 | *Hd1* | |
| DPZ | 11_2020 | **7** | **5.0–16.2** | **RM21137–RM5481** | **10.4** | **-3.1** | **1.7** | **82.9** | *Ghd7* | |
| DPZ | 11_2021 | **7** | **8.8–16.2** | **RM21324–RM5481** | **7.3** | **-3.9** | **1.8** | **25.3** | *Ghd7* | |
| DPZ | 11_2021 | **7** | **28.1–29.4** | **RM22105–RM22164** | **6.6** | **-5.5** | **1.0** | **34.5** | | *OsPRR37* |
| DPZ | 11_2022 | 8 | 5.5–10.3 | RM22550–RM3395 | 10.8 | 6.3 | 5.6 | 89.0 | *DTH8* | |
| DPZ | 11_2032 | **10** | **17.5–20.7** | **RM5620–RM25771** | **5.1** | **2.2** | **2.0** | **59.6** | | *Ehd1* |
| DPZ | 11_2031 | **12** | **1.0–2.3** | **RM3323–RM3747** | **2.1** | **-4.1** | **-0.2** | **19.1** | | |
| DPZ | 11_2023 | 12 | 21.2–26.0 | RM1986–RM1300 | 3.8 | -2.2 | 1.8 | 40.7 | | |

| Population | | Chromo some | Physical position | Marker Interval | LOD[a] | Additive effect[b] | Dominance effect[c] | PVE (%)[d] | Corresponding gene[e] | Located near gene[f] |
|---|---|---|---|---|---|---|---|---|---|---|
| KMK | 10_2095 | **2** | **9.5–15.6** | **RM12921–RM13165** | **6.2** | **-1.3** | **-1.5** | **56.9** | | |
| KMK | 10_2108 | **2** | **26.1–33.0** | **RM13679–RM7286** | **2.1** | **0.8** | **0.7** | **33.5** | | *DTH2* |
| KMK | 10_2095 | 3 | 0.4 | RM7332 | 2.1 | -0.6 | 0.9 | 16.3 | | *Ehd4* , *DTH3* |
| KMK | 10_2090 | 3 | 3.2–9.9 | RM4683–RM1371 | 3.1 | -1.9 | -1.6 | 45.9 | | |
| KMK | 10_2093 | 3 | 32.4–36.2 | RM6970–RM7389 | 16.1 | -9.9 | 2.1 | 92.2 | | *Hd6* , *Hd16* |
| KMK | 10_2096 | 5 | 4.7–6.8 | RM17990–RM18107 | 2.2 | 0.6 | -0.5 | 35.1 | | |
| KMK | 10_2098 | 5 | 23.9–29.8 | RM3476–RM19221 | 4.9 | -3.4 | -9.7 | 53.4 | | |
| KMK | 10_2085 | 5 | 27.9–29.8 | RM5784–RM19221 | 2.7 | -1.2 | -1.0 | 41.0 | | |
| KMK | 10_2099 | **6** | **0.1–8.1** | **RM19227–RM19725** | **3.6** | **-3.5** | **-1.8** | **41.8** | *RFT1, Hd3a* | *Hd17* |
| KMK | 10_2099 | 6 | 17.1–20.3 | RM20084–RM7193 | 2.1 | 2.4 | 0.7 | 12.5 | | |
| KMK | 10_2105 | 7 | 0.1 | RM5911 | 3.1 | 2.5 | 1.2 | 23.7 | | |
| KMK | 10_2088 | **8** | **3.0** | **RM4955** | **5.7** | **-2.5** | **0.6** | **66.5** | | |
| KMK | 10_2105 | **8** | **3.0** | **RM4955** | **5.1** | **-1.3** | **1.4** | **62.7** | | |
| KMK | 10_2106 | 8 | 3.0–8.5 | RM4955–RM22674 | 3.5 | -2.5 | 0.2 | 47.4 | *DTH8* | |
| KMK | 10_2106 | 8 | 19.5–24.6 | RM23068–RM5891 | 2.5 | 1.3 | 1.7 | 17.0 | | |
| KMK | 10_2107 | 8 | 24.6–28.2 | RM5891–RM4997 | 3.3 | 1.0 | -1.5 | 49.0 | | |
| KMK | 10_2094 | 10 | 0.1 | RM7492 | 2.5 | -0.5 | -1.3 | 38.9 | | |
| KMK | 10_2084 | 10 | 11.7–13.6 | RM4455–RM1859 | 3.3 | -0.7 | -0.5 | 49.4 | | |

| Population | | Chromo some | Physical position | Marker Interval | LOD[a] | Additive effect[b] | Dominance effect[c] | PVE (%)[d] | Corresponding gene[e] | Located near gene[f] |
|---|---|---|---|---|---|---|---|---|---|---|
| NAB | 11_2046 | **2** | **34.7** | **RM3789** | **2.1** | **-1.5** | **0.7** | **24.0** | | *DTH2* |
| NAB | 11_2043 | 3 | 0.5–5.5 | RM4108–RM5442 | 2.3 | -4.2 | 0.2 | 19.7 | | *Ehd4* , *DTH3* |
| NAB | 11_2044 | **3** | **9.9–15.0** | **RM1371–RM3204** | **4.8** | **-2.5** | **-0.5** | **61.0** | | |
| NAB | 11_2040 | 3 | 30.4–35.6 | RM3199–RM3329 | 7.4 | -8.5 | 5.4 | 84.0 | *Hd6* | *Hd16* |
| NAB | 11_2045 | 3 | 30.4–35.6 | RM3199–RM3329 | 10.5 | -7.9 | 4.0 | 51.0 | *Hd6* | *Hd16* |
| NAB | 11_2046 | 4 | 2.0–4.9 | RM5414–RM16449 | 2.4 | 2.0 | 0.3 | 29.0 | | |
| NAB | 11_2049 | 5 | 4.2–8.3 | RM3777–RM2744 | 2.4 | -2.3 | -1.2 | 39.0 | | |
| NAB | 11_2051 | **6** | **2.2–6.0** | **RM8112–RM2615** | **3.2** | **-1.6** | **-1.8** | **47.0** | *RFT1, Hd3a* | *Hd17* |
| NAB | 11_2052 | **6** | **2.2–6.0** | **RM8112–RM2615** | **4.5** | **3.6** | **3.8** | **14.0** | *RFT1, Hd3a* | *Hd17* |
| NAB | 11_2052 | **6** | **8.8–15.8** | **RM5963–RM20023** | **6.2** | **8.0** | **-1.8** | **34.0** | *Hd1* | |
| NAB | 11_2067 | **6** | **8.8–20.3** | **RM5963–RM7193** | **14.2** | **8.1** | **-0.6** | **86.0** | *Hd1* | |
| NAB | 11_2043 | 6 | 13.0–23.3 | RM19951–RM1340 | 5.3 | 6.9 | 3.2 | 55.4 | | |
| NAB | 11_2053 | 6 | 13.0–20.3 | RM19951–RM7193 | 8.4 | 5.9 | -0.7 | 79.0 | | |
| NAB | 11_2055 | 7 | 5.7–16.2 | RM6728–RM5481 | 4.0 | -7.0 | -0.3 | 54.0 | *Ghd7* | |
| NAB | 11_2054 | 7 | 5.7–16.2 | RM6728–RM5481 | 7.5 | -5.3 | 1.9 | 75.0 | *Ghd7* | |
| NAB | 11_2055 | 7 | 25.7–29.4 | RM1330–RM22164 | 7.4 | -6.2 | 0.1 | 32.0 | | *OsPRR37* |
| NAB | 11_2045 | **8** | **1.8–5.9** | **RM22321–RM6838** | **7.4** | **-6.9** | **2.2** | **40.0** | *DTH8* | |
| NAB | 11_2056 | **8** | **5.9–12.4** | **RM6838–RM22839** | **9.1** | **-5.6** | **1.3** | **81.0** | *DTH8* | |
| NAB | 11_2057 | **8** | **5.9–18.8** | **RM6838–RM5767** | **5.4** | **-4.6** | **1.8** | **65.0** | *DTH8* | |
| NAB | 11_2037 | 10 | 0.1 | RM7492 | 2.1 | -0.8 | -1.9 | 34.0 | | |
| NAB | 11_2061 | 10 | 18.8–23.1 | RM6737–RM6673 | 2.2 | 2.2 | 1.7 | 35.0 | | |
| NAB | 11_2040 | 12 | 3.8–4.9 | RM27627–RM3455 | 2.2 | -4.4 | -5.6 | 16.0 | | |
| NAB | 11_2067 | 12 | 4.9–16.3 | RM3455–RM28112 | 3.4 | -1.8 | -0.7 | 5.0 | | |

| Population | | Chromosome | Physical position | Marker Interval | LOD[a] | Additive effect[b] | Dominance effect[c] | PVE (%)[d] | Corresponding gene[e] | Located near gene[f] |
|---|---|---|---|---|---|---|---|---|---|---|
| BKH | 10_2046 | 1 | 41.2–42.9 | RM5310–RM8137 | 2.5 | 1.3 | 0.1 | 38.3 | | |
| BKH | 10_2053 | **3** | **0.5** | **RM4108** | **2.9** | **-1.3** | **2.8** | **42.9** | | *Ehd4* , *DTH3* |
| BKH | 10_2068 | **3** | **0.5** | **RM4108** | **5.3** | **-7.8** | **-2.3** | **36.7** | | *Ehd4* , *DTH3* |
| BKH | 10_2049 | **3** | **0.5–9.9** | **RM4108–RM1371** | **5.8** | **-4.6** | **2.2** | **67.0** | | *Ehd4* , *DTH3* |
| BKH | 10_2050 | 3 | 21.4–25.1 | RM5488–RM3513 | 2.4 | -1.8 | -0.9 | 37.6 | | |
| BKH | 10_2051 | 3 | 27.3–32.4 | RM6736–RM6970 | 8.7 | -8.6 | 7.3 | 80.0 | *Hd6* | *Hd16* |
| BKH | 10_2056 | 5 | 0.1–5.9 | RM1248–RM18055 | 4.2 | -3.0 | 0.9 | 25.0 | | |
| BKH | 10_2058 | 5 | 20.1–22.3 | RM1386–RM3295 | 2.7 | 2.3 | 1.9 | 15.2 | | |
| BKH | 10_2058 | **5** | **22.3–27.9** | **RM3295–RM5784** | **4.7** | **-5.2** | **0.1** | **41.9** | | |
| BKH | 10_2056 | **6** | **0.2–5.2** | **RM6467–RM5754** | **5.8** | **-3.2** | **-1.7** | **33.7** | *RFT1, Hd3a* | *Hd17* |
| BKH | 10_2057 | **6** | **0.2–5.2** | **RM6467–RM5754** | **4.9** | **-5.0** | **-3.8** | **18.1** | *RFT1, Hd3a* | *Hd17* |
| BKH | 10_2058 | **6** | **0.2–5.2** | **RM6467–RM5754** | **4.4** | **-4.0** | **0.8** | **36.5** | *RFT1, Hd3a* | *Hd17* |
| BKH | 10_2059 | **6** | **0.2–5.2** | **RM6467–RM5754** | **5.3** | **-7.3** | **-3.5** | **22.2** | *RFT1, Hd3a* | *Hd17* |
| BKH | 10_2071 | **6** | **0.2–5.2** | **RM6467–RM5754** | **4.8** | **-4.8** | **-2.3** | **60.1** | *RFT1, Hd3a* | *Hd17* |
| BKH | 10_2045 | 6 | 0.2–9.0 | RM6467–RM19771 | 6.9 | -11.7 | -4.3 | 68.6 | *Hd1* | |
| BKH | 10_2068 | **6** | **5.2** | **RM5754** | **7.4** | **-4.8** | **-3.5** | **47.0** | *RFT1, Hd3a* | *Hd17* |
| BKH | 10_2073 | **6** | **5.2** | **RM5754** | **4.5** | **-8.7** | **-6.7** | **58.0** | *RFT1, Hd3a* | *Hd17* |
| BKH | 10_2057 | 6 | 5.2–9.0 | RM5754–RM19771 | 7.8 | -9.1 | 4.4 | 35.7 | *Hd1* | |
| BKH | 10_2059 | 6 | 5.2–9.0 | RM5754–RM19771 | 10.2 | -9.4 | 4.5 | 60.7 | *Hd1* | |
| BKH | 10_2075 | 6 | 5.2–9.0 | RM5754–RM19771 | 5.4 | -11.0 | -0.9 | 66.0 | *Hd1* | |
| BKH | 10_2076 | 6 | 5.2–9.0 | RM5754–RM19771 | 9.2 | -15.1 | -5.2 | 81.4 | *Hd1* | |
| BKH | 10_2074 | 6 | 5.2–11.0 | RM5754–RM3330 | 2.9 | -9.7 | -4.4 | 42.9 | *Hd1* | |
| BKH | 10_2060 | 6 | 9.0–20.3 | RM19771–RM7193 | 11.6 | -8.1 | 4.1 | 80.5 | *Hd1* | |
| BKH | 10_2063 | 7 | 5.7–13.4 | RM6728–RM7273 | 4.9 | -4.9 | 0.2 | 60.9 | | |
| BKH | 10_2080 | 7 | 5.7–28.4 | RM6728–RM22120 | 4.5 | 1.4 | 3.0 | 58.1 | | |
| BKH | 10_2062 | **7** | **5.7–23.6** | **RM6728–RM5847** | **7.2** | **-3.6** | **-0.6** | **74.3** | *Ghd7* | |
| BKH | 10_2052 | 7 | 13.4–18.4 | RM7273–RM6394 | 4.2 | -3.4 | 1.1 | 56.1 | | |
| BKH | 10_2065 | 8 | 10.3–14.7 | RM3395–RM22896 | 5.2 | 4.2 | 2.0 | 63.5 | | |
| BKH | 10_2073 | 10 | 17.4–20.7 | RM5620–RM25771 | 4.2 | 0.8 | -7.5 | 55.6 | | *Ehd1* |
| BKH | 10_2076 | 10 | 23.1 | RM6673 | 2.2 | 3.5 | 1.0 | 3.2 | | |
| BKH | 10_2078 | 12 | 5.5–10.1 | RM27723–RM6973 | 2.0 | -0.9 | 1.3 | 36.5 | | |

| Population | | Chromosome | Physical position | Marker Interval | LOD[a] | Additive effect[b] | Dominance effect[c] | PVE (%)[d] | Corresponding gene[e] | Located near gene[f] |
|---|---|---|---|---|---|---|---|---|---|---|
| KNJ | 10_2208 | 1 | 2.2 | RM5423 | 2.1 | -1.4 | 0.1 | 11.5 | | |
| KNJ | 10_2205 | 1 | 14.0–18.7 | RM10850–RM11033 | 2.1 | 0.6 | 0.6 | 24.1 | | |
| KNJ | 10_2194 | 3 | 3.2–7.8 | RM4683–RM14674 | 2.1 | -1.0 | -1.0 | 33.3 | | |
| KNJ | 10_2195 | 3 | 14.5–21.4 | RM6959–RM5488 | 3.2 | -3.4 | -3.2 | 46.7 | | |
| KNJ | 10_2209 | 3 | 25.1–28.7 | RM3513–RM1350 | 2.9 | 1.3 | 0.5 | 20.2 | | |
| KNJ | 10_2193 | 3 | 28.7–32.4 | RM1350–RM6970 | 7.6 | -8.8 | 5.1 | 44.4 | *Hd6* | *Hd16* |
| KNJ | 10_2196 | 3 | 28.7–32.4 | RM1350–RM6970 | 17.9 | -10.8 | 6.7 | 93.2 | *Hd6* | *Hd16* |
| KNJ | 10_2202 | **5** | **20.1–23.9** | **RM1386–RM3476** | **5.2** | **-2.2** | **-1.4** | **63.3** | | |
| KNJ | 10_2203 | **6** | **0.1–16.1** | **RM19227–RM20045** | **5.2** | **-6.9** | **0.8** | **63.4** | *Hd1* | |
| KNJ | 10_2204 | **6** | **6.0–11.8** | **RM2615–RM19882** | **8.8** | **-8.5** | **1.3** | **80.3** | *Hd1* | |
| KNJ | 10_2208 | **7** | **26.8–29.4** | **RM1364–RM22164** | **5.6** | **-2.9** | **1.1** | **66.1** | | *OsPRR37* |
| KNJ | 10_2192 | 8 | 0.1–3.7 | RM6369–RM1148 | 4.0 | -2.3 | 1.3 | 54.1 | | |
| KNJ | 10_2209 | 8 | 0.7–3.7 | RM22258–RM1148 | 5.6 | -2.3 | -0.2 | 57.6 | | |
| KNJ | 10_2210 | 8 | 12.4–16.6 | RM22837–RM22965 | 2.3 | -1.0 | 1.4 | 36.7 | | |
| KNJ | 10_2190 | **9** | **7.4–9.2** | **RM23920–RM1328** | **2.5** | **-1.1** | **0.4** | **39.4** | | |
| KNJ | 10_2214 | 10 | 0.1–9.8 | RM7492–RM1126 | 3.6 | -1.4 | -1.0 | 50.8 | | |
| KNJ | 10_2216 | 11 | 6.3–8.1 | RM4469–RM3701 | 2.7 | 0.8 | -1.2 | 41.3 | | |

| Population | | Chromo some | Physical position | Marker Interval | LOD[a] | Additive effect[b] | Dominance effect[c] | PVE (%)[d] | Corresponding gene[e] | Located near gene[f] |
|---|---|---|---|---|---|---|---|---|---|---|
| BLE | 11_2078 | 1 | 31.5–35.5 | RM5914–RM7594 | 2.6 | -1.6 | 0.0 | 39.8 | | |
| BLE | 11_2080 | 2 | 9.0–13.4 | RM5699–RM13101 | 2.6 | -3.4 | 0.9 | 11.8 | | |
| BLE | 11_2098 | 2 | 35.4 | RM3850 | 2.2 | -1.7 | 0.5 | 35.5 | | *DTH2* |
| BLE | 11_2100 | **3** | **0.5** | **RM4108** | **5.6** | **-5.2** | **-0.6** | **65.7** | | *Ehd4* , *DTH3* |
| BLE | 11_2079 | **3** | **0.5–5.5** | **RM4108–RM5442** | **3.2** | **-2.8** | **0.2** | **25.0** | | *Ehd4* , *DTH3* |
| BLE | 11_2080 | **3** | **26.7–35.6** | **RM2334–RM3329** | **12.7** | **-7.4** | **4.5** | **70.6** | *Hd6* | *Hd16* |
| BLE | 11_2088 | **3** | **30.4–35.6** | **RM3199–RM3329** | **6.5** | **-4.5** | **4.0** | **24.3** | *Hd6* | *Hd16* |
| BLE | 11_2073 | 5 | 0.1–4.2 | RM1248–RM3777 | 2.0 | -1.5 | 0.5 | 32.5 | | |
| BLE | 11_2084 | 5 | 0.1–4.2 | RM1248–RM3777 | 2.6 | -0.7 | 2.7 | 40.4 | | |
| BLE | 11_2085 | 5 | 23.9–27.9 | RM3476–RM5784 | 2.5 | -1.6 | 2.4 | 39.4 | | |
| BLE | 11_2086 | 6 | 0.1–2.2 | RM19227–RM8112 | 2.2 | -2.3 | -1.1 | 48.5 | *RFT1, Hd3a* | *Hd17* |
| BLE | 11_2087 | 6 | 8.8–20.3 | RM5963–RM7193 | 13.5 | -8.1 | 2.3 | 89.8 | *Hd1* | |
| BLE | 11_2088 | **6** | **20.3–23.3** | **RM7193–RM1340** | **5.0** | **-4.1** | **-0.1** | **24.8** | | |
| BLE | 11_2089 | 7 | 2.6–7.1 | RM5752–RM21251 | 2.2 | -1.8 | -0.5 | 35.2 | | |
| BLE | 11_2090 | 7 | 7.1–18.4 | RM21251–RM6394 | 10.2 | -4.5 | 0.8 | 84.2 | *Ghd7* | |
| BLE | 11_2091 | 7 | 29.0–29.4 | RM7601–RM22164 | 7.0 | -4.2 | 3.1 | 73.4 | | *OsPRR37* |
| BLE | 11_2102 | 7 | 29.4 | RM22164 | 5.7 | -3.6 | 1.0 | 70.6 | | *OsPRR37* |
| BLE | 11_2092 | 8 | 0.1–5.9 | RM6369–RM6838 | 10.1 | -6.4 | 1.8 | 83.8 | | |
| BLE | 11_2101 | 10 | 23.1 | RM6673 | 2.7 | 2.2 | -0.5 | 41.9 | | |
| BLE | 11_2103 | 12 | 22.7–24.3 | RM28450–RM28580 | 2.0 | -1.1 | -0.8 | 32.4 | | |

[a] Log-likelihood value. LOD threshold to detect QTLs was determined in each $BC_4F_2$ population.

[b] Additive effect of KSH allele on days to heading

[c] Dominance effect of KSH allele on days to heading

[d] Percentage of phenotypic variance explained by QTL

[e] Previously identified heading date genes corresponding to the QTLs detected in this study based on their physical positions on IRGSP 1.0.

[f] Previously identified heading date genes located near the QTLs detected in this study based on their physical positions on IRGSP 1.0.
