# Supplementary material for: Genetic architecture of variation in heading date among Asian rice accessions
Source: BMC Plant Biol. 2015 May 8;15:115. doi: 10.1186/s12870-015-0501-x (PMC4424449; doi:10.1186/s12870-015-0501-x)
Supplement: Additional file 6Figure S4. — Substitution (boxes) and insertion/deletion (-) polymorphisms of amino acids in the DTH8 protein in 12 diverse accessions of Asian rice. Abbreviations of rice accessions are defined in Table 1. The conserved histon-fold motif domain in DTH8 is indicated in orange. Numbers under the DTH8 diagram indicate the positions of polymorphic sites. Numbers on the right side show the total length of each predicted amino acid sequence. The regions with amino acid changes due to a frame shift are labeled with asterisks. Stop indicates a stop codon. Accession numbers of each sequence are DDBJ: LC016712-LC016721. [file 12870_2015_501_MOESM6_ESM.pdf]

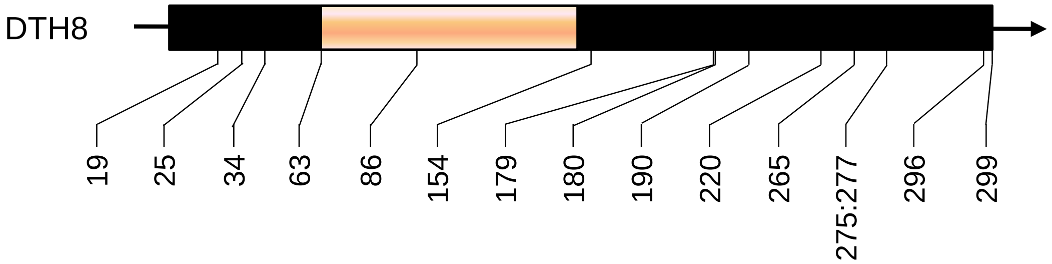

	19	25	34	63	86	154	179	180	190	220	265	275:277	296	299		
KSH	L	E	G	P	S	D	G	-	Y	D	G	3	N	STOP		298
KMK	L	E	G	P	S	D	G	-	Y	D	G	3	N	STOP		298
KNJ	L	E	G	P	S	D	G	-	Y	D	G	3	N	STOP		298
NAB	S	A	G	P	A	A	-	-	S	A	G	3	S	STOP		297
TUP	S	A	G	P	A	A	G	G	S	A	G	3	S	STOP		299
MUH	S	A	G	P	A	A	G	G	S	A	G	3	S	STOP		299
BAS	S	A	G	P	A	A	G	G	S	A	G	-	S	STOP		295
BLE	S	A	G	P	A	A	G	G	S	A	G	-	S	STOP		295
BKH	S	A	G	P	A	A	G	G	S	A	*	*	*	*	STOP	245
QZZ	S	A	G	P	A	A	G	G	S	A	*	*	*	*	STOP	245
DPZ	S	A	G	P	A	A	G	G	S	A	*	*	*	*	STOP	245
HAY	S	A	A	STOP												62

1,118 bp deletion

Figure S4. Substitution (boxes) and insertion/deletion (-) polymorphisms of amino acids in the DTH8 protein in 12 diverse accessions of Asian rice. Abbreviations of rice accessions are defined in Table 1. The conserved histon-fold motif domain in DTH8 is indicated in orange. Numbers under the DTH8 diagram indicate the positions of polymorphic sites. Numbers on the right side show the total length of each predicted amino acid sequence. The regions with amino acid changes due to a frame shift are labeled with asterisks. Stop indicates a stop codon. Accession numbers of each sequence are DDBJ: LC016712-LC016721.
