# Supplementary material for: Genetic architecture of variation in heading date among Asian rice accessions
Source: BMC Plant Biol. 2015 May 8;15:115. doi: 10.1186/s12870-015-0501-x (PMC4424449; doi:10.1186/s12870-015-0501-x)
Supplement: Additional file 7: Table S3. — Allele types of 13 heading date genes isolated by previous studies in 12 diverse accessions of Asian rice. Abbreviations of rice accessions are defined in Table 1. Red and blue characters indicate early heading and late heading alleles, respectively. Numbers, nucleotides, and lower-case letters are respective alleles identified in the previous studies: DTH2 [28]: Ehd4 [31]: DTH3 [26]: Hd6 [43]: Hd16 [29]: Hd17 [27]: RFT1 [15]: Hd3a and Hd1 [33]: Ghd7 [21]: OsPRR37 [32]: DTH8 [25]: Ehd1 [18]. Complete genomic sequences of Hd1, Hd6, Ghd7, Hd3a, RFT1 and DTH8 were determined in the 12 diverse accessions. Functional nucleotide polymorphisms of Ehd1, DTH2, DTH3, Ehd4, Hd17, Hd16 and OsPRR37 were genotyped by gene-specific markers in the 12 diverse accessions. [file 12870_2015_501_MOESM7_ESM.pdf]

Table S3. Allele types of 13 heading date genes isolated by previous studies in 12 diverse accessions of Asian rice. Abbreviations of rice accessions are defined in Table 1. Red and blue characters indicate early heading and late heading alleles, respectively. Numbers, nucleotides, and lower-case letters are respective alleles identified in the previous studies: *DTH2* [28]: *Ehd4* [31]: *DTH3* [26]: *Hd6* [43]: *Hd16* [29]: *Hd17* [27]: *RFT1* [15]: *Hd3a* and *Hd1* [33]: *Ghd7* [21]: *OsPRR37* [32]: *DTH8* [25]: *Ehd1* [18]. Complete genomic sequences of *Hd1* , *Hd6* , *Ghd7* , *Hd3a* , *RFT1* and *DTH8* were determined in the 12 diverse accessions. Functional nucleotide polymorphisms of *Ehd1* , *DTH2* , *DTH3* , *Ehd4* , *Hd17* , *Hd16* and *OsPRR37* were genotyped by gene-specific markers in the 12 diverse accessions.

Accession	*DTH2*	*Ehd4*	*DTH3*	*Hd6*	*Hd16*	*Hd17*	*RFT1*	*Hd3a*	*Hd1*	*Ghd7*	*OsPRR37*	*DTH8*	*Ehd1*
KSH	4	3	6 base deletion	T	A	C	1	1	6	1	1	1	G
HAY	4	3	6 base deletion	T	G	T	1	1	1	4	2	9	G
QZZ	1	3	6 base deletion	A	G	C	1	1	3	4	2	10	G
TUP	1	3	6 base deletion	A	G	C	4	3	17	1	3	4	G
MUH	1	3	6 base deletion	A	G	C	4	3	13	1	3	4	G
BAS	4	2	6 base deletion	A	G	C	1	1	2	1	3	3	G
DPZ	1	3	6 base deletion	A	G	C	4	3	1	3	3	10	G
KMK	4	2	6 base deletion	A	G	C	1	1	12	1	3	1	G
NAB	1	3	6 base deletion	A	G	C	4	3	16	2	3	5	G
BKH	1	3	6 base deletion	A	G	C	2	5	1	2	1	10	G
KNJ	4	2	6 base deletion	A	G	C	1	1	9	1	3	1	G
BLE	1	3	6 base deletion	A	G	C	2	6	4	2	3	3	G
